# Do evaluative statements in facial identification overstate the strength of the evidence?

**DOI:** 10.1111/1556-4029.70265

**Published:** 2026-01-26

**Authors:** Nada Aggadi, Reuben Moreton, Thomas Busey

**Affiliations:** ^1^ Department of Psychological and Brain Sciences, Indiana University Bloomington Indiana USA; ^2^ Reli Southampton UK

**Keywords:** bias, calibration, error rates, face identification evidence, likelihood ratios, ordered probit model, strength of evidence, subjectivity

## Abstract

Facial identification examiners assess whether two facial images—such as an image of an unknown person from surveillance footage and a controlled image of a known individual—depict the same person or different people. To communicate their observations, they rely on predefined verbal articulation scales that sometimes have associated numeric equivalents. However, these terms have not been calibrated against the actual strength of the evidence except indirectly through proficiency tests and black box studies. The present research reanalyzes the findings of face comparisons from the most comprehensive facial identification black box study to date, as well as multiple facial examination proficiency tests, to generate a quantitative measure of the strength of the evidence for each comparison. We used an ordered probit model to summarize the distribution of responses of both individual examiners and examiner teams to produce a set of likelihood ratios for each group and test. The likelihood ratios can be lower than values implied by the evaluative statements, which do not seem to justify the strengths of evidence implied by current articulation scales used in facial comparisons. Our analyses suggest that examiners are using language that overstates the strength of the evidence by several orders of magnitude.

**Highlights**

- We converted black box and proficiency test data into numerical distributions.
- We generate likelihood ratios for individual comparisons from numerical distributions.
- Demonstrates that current verbal scales may overstate the strength of evidence.
- Articulation language should accurately reflect the strength of the evidence.

## 1 INTRODUCTION

Forensic facial examiners (FFEs) provide expert opinion on facial identity and are trained to identify similarities and differences between individual facial features when comparing images such as photographs, surveillance footage, and videos. Facial comparison methods used by FFEs are typically based on morphological analysis. Morphological analysis assesses the shape, morphology, and degree of similarity between facial feature components such as the nose or ear, and their corresponding component characteristics such as symmetry, location, and relative proportion. Examiners may also rely on distinguishing features like scars and tattoos [1]. Morphological analysis is the technique currently recommended by the Facial Identification Scientific Working Group (FISWG) and the European Network of Forensic Science Institutes (ENFSI). Because several factors can make facial comparisons more challenging, such as image quality, appearance changes, ethnicity, or age [2], FFEs undergo extensive training that can last several years [3]. Studies have shown that FFEs are significantly better than untrained participants at facial image comparison tasks and rival the performance of state‐of‐the‐art automated facial recognition algorithms [4, 5, 6, 7].

FFEs evaluate the observations from a facial comparison and express the findings as a strength of evidence for one of two competing propositions, typically a same‐source proposition and a different‐source proposition [8]. In the absence of high‐quality quantitative methods on which to base the examination, FFEs have traditionally communicated their results as a qualitative opinion, using various iterations of verbal scales with terms such as: “Support for different source,” “Support for same source,” “Extremely strong support different people,” or “Extremely strong support same person.”

Although there are no universally accepted scales used by FFEs, there are guidelines that provide recommended wording for the articulation of scales in facial comparison. One such guideline is the *Standard Guide for Image Comparison Opinions* provided by the National Institute of Standards and Technology Organization of Scientific Area Committees (OSAC) [9] and another is the ENFSI *Guideline for Evaluative Reporting in Forensic Science* produced [10]. Both guidance documents are similar in the sense that they recommend the observations from the facial comparison be conveyed as a strength of evidence in support of a same‐source or different‐source proposition, which are evaluative statements rather than definitive conclusions.

The likelihood ratio is a ratio of how likely it would be to make an observation under two competing and mutually exclusive hypotheses or propositions [11]. The likelihood ratio is an important component in the theory of Bayesian belief updating as it allows new information to be combined with existing information [12]. In the case of forensic facial examination, the observations of facial feature similarity and/or dissimilarity would be assessed under the proposition that the images depict the same person (the same source proposition) versus the proposition that the images depict different people selected at random from a relevant population (the different sources proposition). The ratio of these two probability densities is the likelihood ratio, and it is a measure of the relative strength of support for the two propositions. Likelihood ratios are widely used to convey the strength of evidence in forensic science for disciplines where high‐quality quantitative data is available (e.g., DNA analysis). In the absence of quantitative data from which to derive probability density functions, forensic experts may convey the strength of evidence as a qualitative evaluative opinion based on technical knowledge and simulated tests [13]. Typically, these evaluative opinions are expressed using a verbal equivalent of the numerical likelihood ratio, and in some instances, these verbal equivalents are expressly linked to a range of likelihood ratio values (e.g., strong support for a proposition is associated with a likelihood ratio value that ranges from 1000 to 10,000 [14]).

In part due to the lack of quantitative approaches, verbal equivalent scales are widely used in forensic face examination to express the strength of evidence. However, it is unclear whether the scales are well calibrated and effective at conveying the examiner's intended strength of evidence. The approach described in the present work applies a statistical model developed to analyze error rate data from friction ridge comparisons by Busey & Coon [15] and subsequently applied to firearms error rate data by Aggadi, Zeller, & Busey [16] as well as palmprint data by Coon and Busey [17]. This approach creates a quantitative measure of a set of verbal evaluative opinions in the form of a likelihood ratio, which converts words to numbers and creates a scale that directly expresses the strength of the evidence. Details about the model's structure, assumptions, and validation can be found in the three earlier works, where the technique was developed and rigorously evaluated. Rather than reintroducing the model in full, this paper focuses on extending its application to the domain of facial identification—an area where evaluative statements have been adopted without consistent empirical calibration and validation. Below, we provide a brief summary of this model, and in Supporting Information, we provide a more extensive description of the approach for those readers who are unfamiliar with the model and want additional information about the model architecture and assumptions.

## 2 THE ORDERED PROBIT MODEL

**FIGURE 1:**
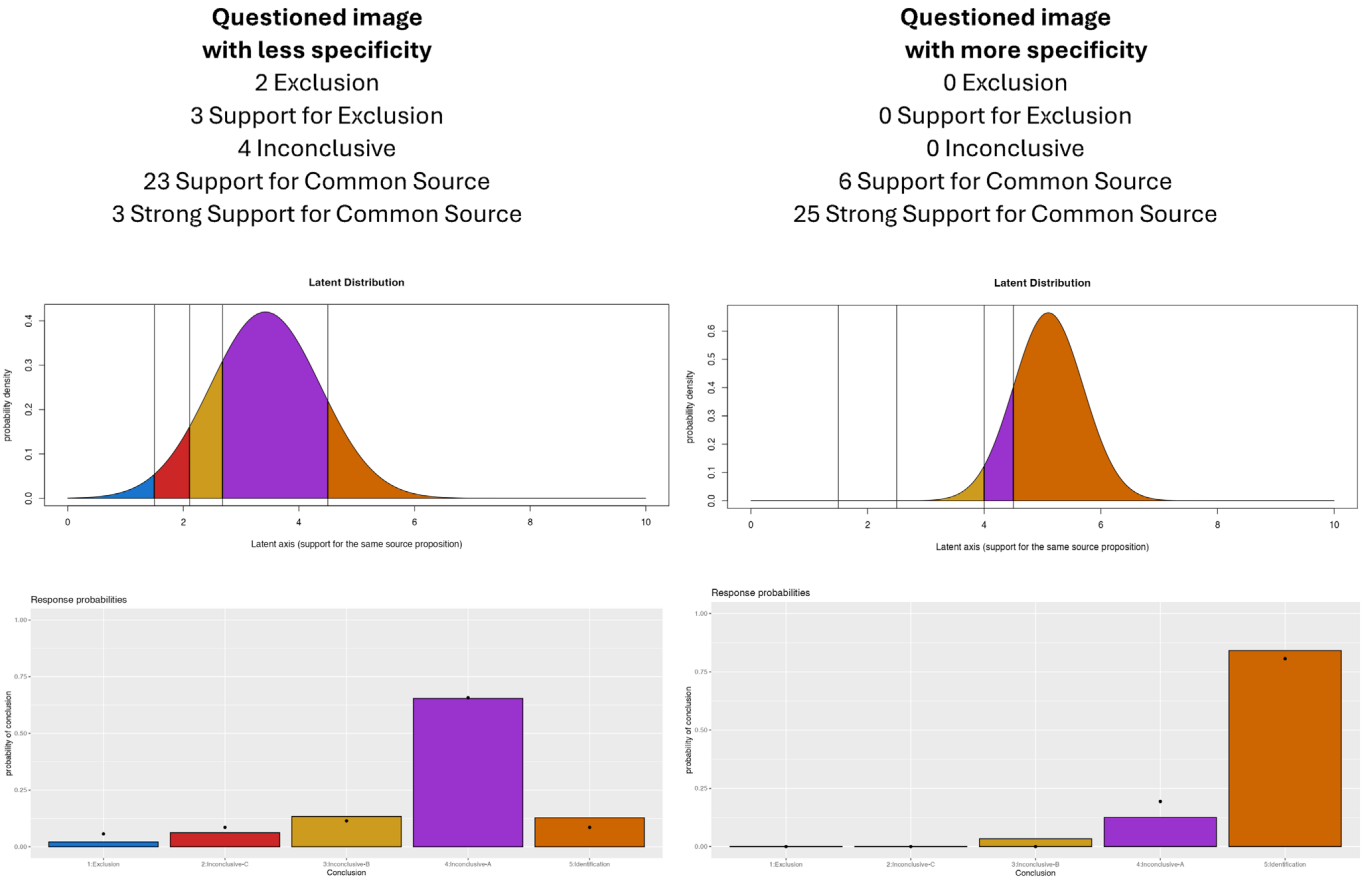
Left Column: Illustration of an ordered probit model applied to face identification response probabilities (black dots in lower panel) with the best fitting parameters. In this case, the questioned image has less specificity (e.g., poor image quality or partially occluded), leading to few “Strong Support for Common Source” determinations and a normal distribution shifted to the left. This produces a set of predictions that roughly corresponds to the empirical frequency distribution (dots in lower graph). Right column: Illustration of an ordered probit model applied to face identification response probabilities (black dots in lower panel) with the best fitting parameters. In this case, the questioned image has more specificity (e.g., high image quality and no occlusion), leading to many “Strong Support for Common Source” determinations. The model responds to this shift by moving the normal distribution to the right to make the colored bars align with the empirical data (black dots in the lower graph).

The ordered probit model summarizes black box data in such a way that likelihood ratios can be created based on the verbal responses made by facial identification examiners. This approach translates the distribution of verbal responses into a strength of support on a latent scale, where larger values on this scale provide more support for the same source proposition and smaller values provide less support for the same source. The distribution of responses comes from the combined responses of all FFEs who complete a comparison in a proficiency or error rate test. Thus, we rely on the entire distribution of responses rather than creating a likelihood ratio associated with a particular verbal response from a scale. The left panel of Figure 1 illustrates a response distribution from a pair where the questioned face contained less specificity and detail, with FFE responses ranging from “Exclusion” to “Strong Support for Common Source.” In the right column of Figure 1, the model is applied to a questioned face with more specificity and detail, where a majority of FFEs reached a “Strong Support for Common Source” decision. The graphs in the lower row of Figure 1 show the response frequencies as summarized by normal distributions as predicted by the ordered probit model, using parameters specific to each face comparison. The left column of Figure 1 has a normal distribution shifted to the left to reflect the wide range of responses across FFEs, while the right column has a normal distribution shifted to the right to reflect the large number of “Strong Support for Common Source” responses. To help readers better understand how the model operates, we have created an interactive demonstration that allows users to experiment with different parameter values and observe how shifts in the underlying normal distribution affect the predicted pattern of verbal responses: https://iupbsapps.shinyapps.io/OrderedProbitDemoFacialIdentification/.

The ordered probit model estimates the mean (*μ*) and standard deviation (*σ*) of the normal distribution along the latent axis for each image pair, along with all but two of the thresholds that partition the latent axis into verbal response categories. There are several more steps to convert these latent axis distributions to likelihood ratios, but first, we describe the black box study and proficiency tests we analyzed using the model. All analysis code and the datasets used are available on OSF.io at the following link: https://osf.io/j2w49/?view_only=e9eaa514f96249a0b045fc22bd43eb68.

## 3 APPLICATIONS TO BLACK BOX AND PROFICIENCY TEST STUDIES

### 3.1 National Institute of Standards and Technology

We analyzed data collected by Phillips, Yates [5], who compared the face identification accuracy of forensic experts, super‐recognizers, students, and machine comparison systems. Participants were asked to judge pairs of face images using a 7‐point confidence scale ranging from “strong support for same person” (+3) to “strong support for different person” (−3). Examiners and reviewers completed comparisons in their labs with professional tools, while super‐recognizers and fingerprint examiners used standard computer tools; students completed the task on a preset computer interface. Stimuli were selected from over 9000 images of 507 individuals, and subsets were chosen using both algorithmic difficulty rankings and prior human performance data.

### 3.2 The ENFSI

Proficiency tests are a requirement for accreditation to the ISO/IEC 17025:2017 standard and gather data on organizational performance. In the current study, we are using data from proficiency tests from three different test providers. The first is the ENFSI. The ENFSI tests compared the ability of single examiners, untrained individuals from the general population, and teams of examiners to analyze and compare pairs of facial images. We used data from the 2018 and 2022 ENFSI proficiency tests. The 2018 ENFSI proficiency test was the first published test to compare the performance of professional teams of examiners and individual FFEs. The 2022 test focused specifically on individuals of different ages but also compared the performance of individual FFEs and professional teams. The analysis of professional teams using the ordered probit model is provided in Supporting Information. These yearly tests are designed around specific themes to evaluate a broad range of forensic image comparison skills across varying contexts. The proficiency tests both consisted of 20 challenging one‐to‐one face comparisons designed to be representative of forensic casework. All participants responded using an 11‐point scale from −5 (*Extremely strong support different people*) to +5 (*Extremely strong support same person*), where the midpoint 0 indicates the comparison provides “inconclusive” evidence for either determination. The scale also includes equivalent numerical likelihood ratio ranges for each verbal statement. For more details about the test designs, please refer to Towler, Dunn [7] for the 2018 test and Sexton, Moreton [6] for the 2022 test.

The expanded 11‐point scale is easily accommodated by the ordered probit model. We now have 10 thresholds to separate the latent dimension into 11 response categories. The outer two are fixed to set the scale, and the inner eight are estimated. Each comparison has 10 degrees of freedom and only two estimated parameters (*μ* and *σ*), so the model is very far from saturated even accounting for the 8 estimated thresholds that are common to all participants and image pairs.

### 3.3 Ideal innovations incorporated [18]

Ideal innovations incorporated (I3) is a US‐based forensic and biometric company and was an accredited provider of facial identification proficiency tests from 2021 to 2023. The I3 tests compared the performance of individual examiners to analyze and compare pairs of facial images. We used data from the 2021, 2022, and 2023 tests and combined the responses to improve the overall likelihood ratio estimates. The summary reports for these tests are publicly available from the I3 website. Similar to the ENFSI tests, each I3 test comprised 20 one‐to‐one face comparisons designed to be representative of forensic casework. All participants responded using a five‐point verbal scale from *Exclusion* to *Strong support for common source*. There is no additional information available as to whether the I3 tests conformed to a specific theme or type of imagery.

### 3.4 Collaborative testing services [19]

Collaborative testing services (CTS) are a US‐based accredited provider of proficiency testing services and provide a facial identification proficiency test in collaboration with I3. The test is similar in format to previous I3 tests, except that the five‐point scale now ranges from *Strong support for different source* to *Strong support for common source*.

## 4 ORDERED PROBIT LIKELIHOOD RATIOS

Ordered probit likelihood ratios are calculated based on the initial distribution of examiner responses for each comparison pair, and in this section, we describe several examples and then summarize the steps needed to convert each response distribution into a likelihood ratio. The right‐hand columns of Table 1 summarize data collected for the Facial examiners from the NIST data, with each row indicating how many examiners assigned each level of support to a specific pair. For example, in comparison 04900d172_04900d155, responses included 0 examiners indicating Strong Support for the same person, 2 indicating Support for the same person, 19 marking support to some extent that it is the same person, 22 stating that the observations support neither that it is the same person nor that it is different persons, 8 indicating that the observations support to some extent that it is not the same person, 4 examiners marking the observations as support that it is not the same person, and 2 for whom the observations strongly support that it is not the same person. In contrast, for comparison 05232d114_05232d1563_2022 (right column of Figure 1) we have 28 examiners indicating Strong Support for the same person, 20 indicating Support for the same person, 4 marking support to some extent that it is the same person, 1 examiner stating that the observations support neither that it is the same person nor that it is different persons, 2 indicating that the observations support to some extent that it is not the same person, 2 examiners marking the observations as support that it is not the same person, and none for whom the observations strongly support that it is not the same person. These two distributions reflect markedly different levels of support for the same source proposition, illustrating how examiner response distributions can vary across different image pairs.

**TABLE 1:** Data from the Facial examiners from the NIST data. We calculated the *μ* and *σ* values using the ordered probit model, and sorted the pairs from the lowest *μ* to the highest *μ*. The numbers on the right side of the table represent the number of examiners who responded with “strongly support that it is not the same person” (−3), “support that it is not the same person” (−2), and “support to some extent that it is not the same person” (−1), “Support neither that it is the same person nor that it is different persons” (0), “Support to some extent that it is the same Person” (+1), “support for same person” (+2), and “strong support for same Person” (+3). Each pair's ground truth is indicated by the column “Mated,” with False referring to nonmated (different source) pairs and True referring to mated (same source) pairs.

PairID	Mated	*μ*	*σ*	LR	m3	m2	m1	zero	p1	p2	p3
04628d459_05252d168	FALSE	1.43	1.65	0.07	28	19	7	0	1	1	1
04509d590_05152d73	FALSE	1.98	1.64	0.14	19	24	9	0	0	4	1
05041d80_04986d102	FALSE	2.35	1.57	0.21	14	23	11	2	2	4	1
04605d377_05017d229	FALSE	2.70	1.51	0.31	12	17	9	5	11	3	0
05115d184_04778d77	FALSE	2.74	1.12	0.32	10	11	17	15	4	0	0
04605d295_05040d47	FALSE	2.75	1.72	0.32	11	20	13	0	4	8	1
04884d76_05180d63	FALSE	2.81	0.91	0.34	4	19	19	13	1	1	0
04670d374_04738d125	FALSE	2.97	1.27	0.41	6	17	16	4	11	3	0
04900d172_04900d155	TRUE	3.69	0.91	0.89	2	4	8	22	19	2	0
04937d81_04937d171	TRUE	3.90	1.35	1.11	4	4	9	8	25	5	2
05113d201_05113d21	TRUE	4.38	1.14	1.89	1	3	4	9	25	14	1
04379d484_04379d636	TRUE	4.46	1.36	2.04	1	6	6	2	22	18	2
05244d182_05244d77	TRUE	4.67	1.25	2.60	0	3	6	5	22	17	4
04512d600_04512d777	TRUE	4.78	1.36	2.92	0	3	8	2	21	17	6
05036d128_05036d43	TRUE	5.38	1.69	5.89	1	1	5	5	12	18	15
04948d250_04948d133	TRUE	6.00	1.12	12.29	0	0	0	1	10	28	18
04876d123_04876d332	TRUE	6.16	1.48	14.90	1	0	0	1	9	24	22
05232d114_05232d156	TRUE	6.49	1.77	22.20	0	2	2	1	4	20	28
04297d338_04297d462	TRUE	6.57	2.12	24.76	2	1	0	2	8	14	30
04237d170_04237d284	TRUE	6.64	1.53	26.74	0	1	0	1	5	20	30

**FIGURE 2:**
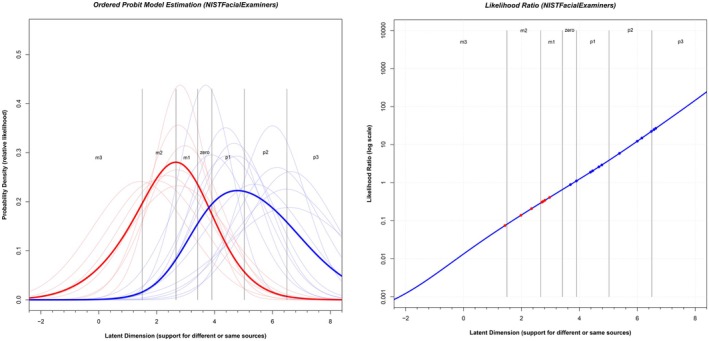
Left panel: Relative likelihood of observing a given latent value for each mated (light blue curves) or nonmated (light red curves) comparison for the Facial examiners from the NIST data. The parameters for each normal distribution were derived from the ordered probit model fit to all seven conclusions for each comparison. The thick red curve corresponds to the sum of light red curves. It represents the relative likelihood of observing any nonmated comparison at each value of the latent axis. The thick blue curve represents the relative likelihood of observing any mated comparison at each value of the latent axis. The vertical lines correspond to the median threshold values that divide the latent axis to produce the estimate of the proportion of responses in each bin. The labels “m3” correspond to −3 on the response scale, while “p3” corresponds to 3 on the response scale. Right panel: Likelihood ratio values for different values along the latent axis for the Facial examiners from the NIST data. The y axis is plotted on a log (10) axis. Likelihood ratios for individual mated pairs are shown as blue circles, and likelihood ratios for nonmated pairs are shown as red circles.

The left panel of Figure 2 displays the full set of normal distributions defined by their respective *μ* and *σ* parameters for each image pair compared by facial examiners in the NIST data. *The right endpoint of the latent axis corresponds to the “most support for the same sources proposition” and the left endpoint of the latent axis corresponds to the “most support for the different sources proposition.” The right endpoint of the latent axis corresponds to the “most support for the same sources proposition and the left endpoint of the latent axis corresponds to the ‘most support for the different sources proposition’.”* Each light red curve represents the latent distribution associated with a different source pair, while each light blue curve corresponds to the same source pair. The variation in the location of these curves along the latent axis demonstrates that the image pairs differ in the degree of support given for each proposition. The height of each curve reflects the probability density of the normal distribution—that is, the likelihood that an examiner would reach a specific latent value when evaluating that image pair.

Two additional steps are required to compute likelihood ratios from the output of the ordered probit model.

The first step involves generating overall probability curves that summarize the responses across all pairs. In Figure 2, the thin curves represent the probability of observing a particular latent value given a specific image pair. However, to calculate a likelihood ratio, we need the probability of each latent value across *any* same source pair and *any* different source pair. To achieve this, we assume the pairs are independent and apply the probability “or” rule—summing the normal distributions for all same source pairs and normalizing the total area to 1.0. This results in the thick blue curve in Figure 2, which represents the distribution of latent values for any given source pair. We repeat this process for different source pairs, generating the thick red curve. The likelihood ratio at each point along the latent axis is then computed as the ratio of the blue to red curves, shown in the right panel of Figure 2. As with the left panel of Figure 2. Again, the right endpoint of the latent axis corresponds to the “most support for the same sources proposition" and the left endpoint of the latent axis corresponds to the "most support for the different sources proposition." These likelihood ratios increase with larger values of the latent axis, which is expected: as more examiners indicate “Support for Same Person,” the support for the same source proposition increases. When plotted on a log scale, this relationship appears roughly linear—an outcome that is reasonable but not guaranteed.

In the second step, we compute the likelihood ratio for each individual image pair. To do this, we locate the *μ* value for that image pair on the right side of Figure 2 to determine the corresponding ordered probit likelihood ratio for that image pair. To do this, we locate the *μ* value for that image pair on the right side of Figure 2 to determine the corresponding ordered probit likelihood ratio for that image pair. For more details, see Busey and Coon [15], Aggadi and Zeller [16], Busey and Coon [17], or the Supporting Information.

Table 1 presents the likelihood ratios for the facial examiners from the NIST data. In this table, rows are sorted by the *μ* value, with different source pairs that received mostly “strong support that it is not the same person” responses at the top. Pairs lower down have more inconclusive responses, and pairs toward the bottom have more “strong support that it is the same person” responses. Consequently, the lower rows tend to have larger *μ* values. A likelihood ratio of 1 implies equal support for the two propositions. Values larger than 1 correspond to more support for the same source of proposition and values less than 1 correspond to more support for different source of proposition. The ordered probit likelihood ratios for same source pairs range from 0.89 to 26.63 and are associated with *μ* values ranging from 3.90 to 6.63. For different source pairs, the ordered probit likelihood ratios ranged from 0.07 to 0.40 and were associated with *μ* values ranging from 1.43 to 2.97.

Table 2 provides the likelihood ratios for the 2018 and 2022 ENFSI data, along with the fitted parameters and response distributions. The ordered probit likelihood ratios for the same source pairs range from 0.79 to 49.53 and are associated with *μ* values ranging from 5.58 to 10.68. For different source pairs, the ordered probit likelihood ratios ranged from 0.79 to 0.11 and were associated with *μ* values ranging from 5.58 to 2.28. Table 2 also contains an additional column, “Proportion Examiners Choosing a Higher Verbal Equivalency Than The LR for LR >1,” which represents the proportion of examiners who overstate the LR. The verbal equivalency value that the original response scale would have applied to that comparison. As we can see for the mated pair in Trial 16, 93% of examiners used a verbal equivalency higher than the likelihood ratio of 49.49. The median proportion of examiners who use a verbal evaluative statement greater than the computed likelihood ratio is 0.75, demonstrating a clear tendency to use language that implies greater support for the same source proposition than the ordered probit likelihood ratio for that image pair. We obtained this equivalency by determining the value of *μ* relative to the various thresholds and associated a verbal label to that value of *μ* using the scale provided by the testing service. For example, Trial 2 in Table 2 has a likelihood ratio of 10.43. The *μ* value is 8.95, which places it between the P3/P4 and P4/P5 thresholds. The associated verbal equivalency provided by the testing service for P4 is 100–10,000, which is the label placed in the verbal equivalency column for Trial 2. Note that this range is much higher than the ordered probit likelihood ratio of 10.43. We see similar miscalibrations across the top and bottom ends of Table 2.

**TABLE 2:** Data from the 2018 and 2022 European Network of Forensic Science Institutes investigation for Examiners. PairIDs starting with “Q” correspond to the 2018 ENFSI data while pairIDs starting with “Trial” correspond to the 2022 ENFSI data. We calculated the *μ* and *σ* values using the ordered probit model, and sorted the pairs from the lowest *μ* to the highest *μ*. The numbers on the right side of the table represent the number of examiners who responded with “Extremely Strong Support for Different People” (m5), “Very Strong Support for Different People” (m4), “Strong Support for Different People” (m3), “Support for Different People” (m2), and “Weak Support for Different People” (m1), “Inconclusive” (zero), “Weak Support for Same Person” (p1), “Support for Same Person” (p2), “Strong Support for Same Person” (p3), “Very Strong Support for Same Person” (p4), and “Extremely Strong Support for Same Person” (p5). Each pair's ground truth is indicated by the column “Mated,” with False referring to nonmated (different source) pairs and True referring to mated (same source) pairs. The “Proportion Examiners Choosing a Higher Verbal Equivalency Than The LR for LR>” column measures the proportion of examiners who use a verbal equivalency statement that is greater than the likelihood ratio generated by the model for a particular image pair. For example, we see that for pair Q1‐m, 75% of examiners chose a higher verbal equivalency than the likelihood ratio generated by the model for this pair. We observe a significant disconnect between the ordered probit likelihood ratios and their verbal equivalence. For example, pair Q18‐m has a likelihood ratio of 8.90, which is associated with a verbal equivalency ranging from 100 to 10,000. See text for more details.

PairID	Mated	*μ*	*σ*	LR	Proportion of examiners choosing a higher verbal equivalency than the LR for LR > 1	m5	m4	m3	m2	m1	Zero	p1	p2	p3	p4	p5
Q3‐nm	FALSE	2.28	1.60	0.11		4	3	7	2	0	0	0	0	0	0	0
Q4‐nm	FALSE	2.67	1.73	0.14		3	4	5	2	1	1	0	0	0	0	0
Q2‐nm	FALSE	2.72	1.66	0.14		2	6	3	4	0	1	0	0	0	0	0
Trial 10	FALSE	3.47	1.56	0.22		5	10	12	23	7	0	2	0	1	0	0
Trial 1	FALSE	3.86	2.00	0.27		7	5	16	14	5	1	6	4	2	0	0
Trial 13	FALSE	4.11	1.56	0.31		1	7	12	21	9	3	2	5	0	0	0
Q6‐nm	FALSE	4.11	1.99	0.31		2	2	1	4	2	3	1	0	1	0	0
Trial 4	FALSE	4.28	1.63	0.34		2	1	19	16	9	2	6	4	1	0	0
Trial 18	FALSE	4.50	1.61	0.39		1	4	11	17	9	7	6	4	1	0	0
Trial 9	FALSE	4.57	1.21	0.41		0	2	8	20	14	11	4	1	0	0	0
Q13‐nm	FALSE	4.68	1.78	0.44		0	1	3	5	1	3	1	1	1	0	0
Trial 7	FALSE	4.79	1.91	0.47		4	3	5	13	12	11	3	4	5	0	0
Trial 3	FALSE	4.90	1.94	0.50		2	3	9	14	12	5	2	6	7	0	0
Trial 17	FALSE	4.92	1.89	0.51		0	4	8	22	9	0	4	6	6	1	0
Q9‐nm	FALSE	5.30	1.90	0.65		1	0	2	2	1	4	2	3	1	0	0
Trial 11	FALSE	5.31	2.18	0.66		2	3	7	11	12	5	6	4	6	3	1
Trial 12	TRUE	5.58	1.90	0.79		1	3	9	4	2	8	16	11	6	0	0
Trial 14	TRUE	5.58	1.53	0.79		0	2	4	7	8	13	13	12	0	1	0
Q14‐nm	FALSE	5.60	1.70	0.80		0	0	0	6	1	4	1	2	2	0	0
Q15‐m	TRUE	5.70	1.91	0.85		0	1	2	2	0	3	2	4	2	0	0
Trial 15	TRUE	5.70	1.96	0.85		2	2	6	2	11	8	11	9	8	1	0
Q12‐m	TRUE	6.24	1.67	1.24	0.56	0	0	1	1	1	4	2	5	2	0	0
Trial 5	TRUE	6.40	1.74	1.39	0.67	1	0	4	3	4	8	11	17	11	1	0
Q7‐m	TRUE	6.44	2.32	1.43	0.62	1	0	1	4	0	0	0	6	1	2	1
Trial 6	TRUE	6.46	2.02	1.46	0.65	2	0	2	3	6	8	16	9	10	1	3
Q16‐m	TRUE	6.56	2.14	1.57	0.69	1	0	0	2	0	2	4	3	2	1	1
Q11‐m	TRUE	6.65	1.85	1.68	0.81	0	0	1	1	0	1	7	3	2	0	1
Trial 8	TRUE	7.32	1.89	2.76	0.57	0	0	1	4	4	2	15	9	14	9	2
Q10‐m	TRUE	7.60	1.64	3.43	0.69	0	0	0	0	0	1	4	5	3	3	0
Q20‐m	TRUE	7.66	1.74	3.61	0.69	0	0	0	1	0	0	4	3	5	3	0
Q19‐m	TRUE	7.97	1.92	4.59	0.69	0	0	0	0	1	1	3	3	4	2	2
Trial 19	TRUE	8.07	2.15	5.00	0.83	0	1	4	0	3	1	1	13	16	17	4
Trial 20	TRUE	8.53	2.29	7.29	0.78	0	0	2	2	3	2	4	7	16	12	12
Q18‐m	TRUE	8.76	2.06	8.88	0.81	0	0	0	0	0	0	3	5	2	1	5
Q5‐m	TRUE	8.87	1.93	9.70	0.94	0	0	0	0	0	1	0	5	4	2	4
Trial 2	TRUE	8.95	2.00	10.42	0.78	1	0	0	1	0	0	5	6	18	19	10
Q8‐m	TRUE	9.00	1.68	10.88	0.81	0	0	0	0	0	0	0	3	8	2	3
Q17‐m	TRUE	9.11	1.91	11.98	0.75	0	0	0	0	0	0	1	3	7	0	5
Q1‐m	TRUE	9.40	1.81	15.39	0.75	0	0	0	0	0	0	0	4	3	5	4
Trial 16	TRUE	10.68	2.00	49.50	0.93	0	0	0	0	1	0	1	2	9	15	32

We also computed likelihood ratios for the 2024 CTS investigation, which can be found in Supporting Information. The ordered probit likelihood ratios for the same source pairs ranged from 1.82 to 506 and were associated with *μ* values ranging from 3.85 to 6.91. For different source pairs, the ordered probit likelihood ratios ranged from 0.04 to 0.01 and were associated with *μ* values ranging from 2.23 to 1.46.

Similarly, we analyzed data from the 2021, 2022, and 2023 I3 Proficiency tests, which can be found in Supporting Information. The ordered probit likelihood ratios for the same source pairs range from 4.04 to 264 and are associated with *μ* values ranging from 3.23 to 6.09. For different source pairs, the ordered probit likelihood ratios ranged from 0.0027 to 0.91 and were associated with *μ* values ranging from 0.21 to 2.91.

## 5 MODEL ASSUMPTIONS AND EVALUATION OF THE NORMAL DISTRIBUTION

A core assumption of the ordered probit model is the normal (Gaussian) distribution, and in this section, we provide a justification for this assumption. The ordered probit model is a generalization of Signal Detection Theory, which is one of the major theoretical frameworks of the last 60 years [20]. The concept of a latent variable space is a core assumption of both signal detection theory and the ordered probit model. The scale of this latent space is set by fixing two parameters; in SDT, this is typically the mean and standard deviation of the noise distribution, but in our approach, we fix the outermost thresholds of the scale in question. This does not make the scale infinitely flexible; indeed, because we compute the likelihood ratio as the ratio of the overall same source and different source distributions, the likelihood ratios are robust against *any* monotonic transformation of this underlying latent axis. The assumption of the Gaussian distribution has a long history of application in science, dating from Fechner [20], and follows directly from assumptions of the Central Limit Theorem. We do explore the *t*‐distribution in the Supporting Information, but treat the Gaussian distribution as the default model. Other distributions might produce slightly different results, but all distributions will be constrained by the data provided by the subjects during testing, which, as we discuss below, demonstrate a fair amount of empirical overlap of the distributions from same source and different source image pairs. No variation of the model produced systematically larger likelihood ratios.

The normal distribution assumption is difficult to test directly, but the critical portion of the different source distribution (the thick red curve in Figure 2) is the right tail. The height of this right tail is the denominator of the likelihood ratio, and the likelihood ratio is very sensitive to this value. If we overestimate it (by having a normal distribution that pushes too much area into the right tail), then our likelihood ratios will underestimate the true strength of the evidence.

**FIGURE 3:**
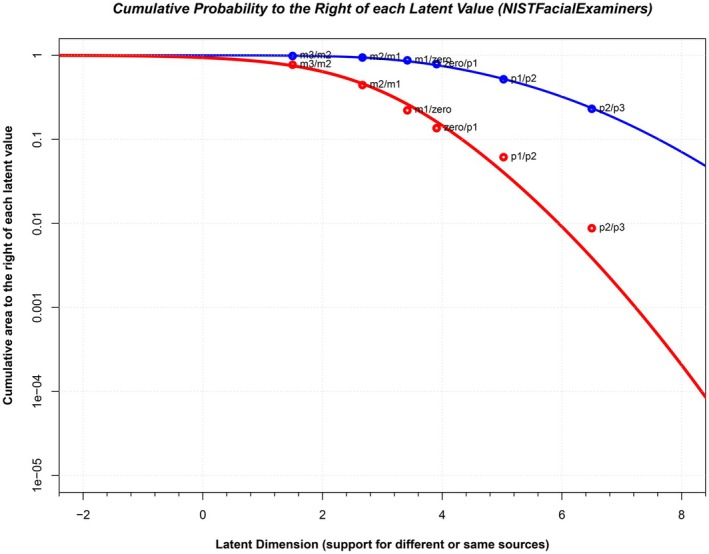
Posterior predictive check of the area to the right of the mated (blue) and nonmated (red) distributions from Figure 2. The continuous curves plot the area to the right of each value along the latent axis under the thick blue and red curves from Figure 2, which represents the right‐hand tail of each distribution. The points represent the empirical proportion of responses at each threshold for mated and nonmated pairs. The close correspondence between the points and curves suggests that the normal distribution is a reasonable summary of the empirical data, with the exception of the right tail of the nonmated distribution (red curve). In this case, we place too little area in the right tail, which will tend to *overestimate* the likelihood ratio (because the denominator is too small). Thus our likelihood ratios, which are already modest relative to the language used by examiners, may actually overestimate the true strength of the evidence.

We can compute the area to the right of individual thresholds as determined by the model and compare those against the empirical values computed directly from examiner responses. Figure 3 illustrates this comparison for the NIST Examiner data. The figure represents a posterior predictive check by comparing the area to the right of the mated (blue) and nonmated (red) distributions from Figure 2 at each point along the latent axis to the proportion of responses for same source and different source pairs. The critical value is the height of the different source (red) curve at each threshold. These values can be compared with the empirical cumulative proportions of responses (shown as red circles) to determine whether the model is under‐ or overestimating the empirical proportions. As can be seen in Figure 3, the points are very close to the curve, which suggests that the normal distribution is doing a reasonable job of extrapolating into the tails of the distribution, with the possible exception of the right tail of the nonmated distribution. In this case, we place too little area in the right tail of the nonmated distribution, which will tend to overestimate the likelihood ratio (because the denominator is too small). Thus, we do not believe that the normal distribution assumption is producing an underestimate of the true strength of the observations. A similar graph can be found for the ENFSI datasets in the Supporting Information, which show a very close correspondence between the empirical and fitted curves.

The summing of the normal distributions to create the overall mated and nonmated pairs in the left panel of Figure 2 requires the assumption of independence of the image pairs [21]. It is possible to approximate these curves by fitting the overall data instead. To do so, we aggregated the raw scores across all mated and nonmated pairs and fit essentially a signal detection theory model with fixed outer thresholds and 4 freely fit thresholds. This produces one nonmated and one mated curve, which are quite similar to the curves in Figure 2. The likelihood ratio for the mated pairs was also very close to the original data with a likelihood ratio for mated pairs of 4.6, which is similar to the median likelihood ratio of 4.4 from Table 1. Thus, we believe that the fits to the aggregate data are quite similar to the fits to individual image pairs that are then summed to create overall mated and nonmated distributions, and the low values we observe are not an artifact of the summing of the normal distributions.

The figures associated with the aggregated analysis can be found in Supporting Information. Similarly, Tables and Figures associated with the ENFSI, I3, and CTS data can be found in Supporting Information. The Supporting Information section also includes sensitivity analyses to show that the likelihood ratios reported in this paper are not a result of particular assumptions. In all cases, the changes in either distribution shapes or Bayesian model parameters either produced lower likelihood ratios or a mixture of higher likelihood ratios for the high and low end, with reduced likelihood ratios (relative to the default assumptions) for likelihood ratio values close to 1. These modest likelihood ratios are the ones that tend to benefit the most from quantification.

## 6 INTERPRETING LIKELIHOOD RATIOS

In Table 1, we can see that for pair 04237d170_04237d284, a majority of examiners have used Strong Support for Same Person when the LR calculated for that pair is around 26.63. According to the ENFSI scale, however, the category “Strong Support for Common Source” is typically associated with LRs ranging from 10,000 to 1,000,000. Whilst the NIST scale does not explicitly state an associated range of LRs, we consider it a good indicator of the intended strength of evidence. The discrepancy between the calculated LR and the indicative intended strength of evidence highlights a disconnect between the computed likelihood ratios and the verbal expressions used by examiners.

Similarly, looking at Table 2, we observe a significant disconnect between the likelihood ratios and the proportion of examiners who overstate the LR in relation to the verbal equivalence in the ENFSI tests. For example, pair Q18‐m has a likelihood ratio of 8.90, but 81% of examiners chose a higher verbal equivalency than 8.90. There is a wide discrepancy between this range and 8.90. Similarly, pair Trial 16 from Table 2 has a likelihood ratio of 1.46, but 65% of examiners chose a higher verbal equivalency than that. While we might expect some examiners to deviate in their strength of evidence, across all image pairs with likelihood ratios greater than 1, all of the values in Table 2 are greater than 0.5, which demonstrates a tendency among examiners to use verbal equivalence statements that are higher than the ordered probit likelihood ratio. In addition to the ENFSI scale, there are other articulation scales with different LR ranges associated with verbal equivalents, such as that proposed by the Association of Forensic Science Practitioners (AFSP) [14]. However, even when we consider the AFSP scale, the range of likelihood ratios across all of the data analyzed (including proficiency tests provided by I3 and CTS) is far different than the verbal equivalency statements provided by AFSP [14], in which strong support for common source implies a likelihood ratio of 1000–10,000. Our data suggest that there needs to be further consideration on what constitutes a calibrated verbal scale. We direct the reader to the Supporting Information for supporting analyses.

Morrison [21] provided a thoughtful commentary on our work [16] in promoting the likelihood ratio framework within the firearms discipline. He noted, however, that while our ordered probit approach represents an important step toward quantitative interpretation of examiner decisions, our model is trained on pooled data from multiple examiners and may not accurately represent the performance of individual examiners. In response, we published a reply [22] clarifying that our primary goal was not to produce examiner‐ or case‐specific likelihood ratios, but rather to evaluate the correspondence between verbal conclusion scales and the strength of evidence observed in black box studies. However, the lack of independence between image pairs in a study (due to the fact that each examiner completed many comparisons in the study) is an important issue, and the solution provided by Morrison [21] is a step in the right direction.

Indeed, it is important to note that the likelihood ratios generated in this study are specific to the testing conditions under which the data were collected. Proficiency tests and black box studies may differ in important ways from specific types of casework and are unlikely to reflect all types of casework imagery [23, 24, 25, 26, 27]. When assessing error rates in forensic disciplines, we should acknowledge the limitations of proficiency tests such as those administered by CTS, I3, and ENFSI. While these tests serve various purposes, they may not reflect the range of image quality experienced during forensic casework. As CTS itself cautions, their test results should not be used to infer accuracy rates among forensic examiners [28]. In addition, some tests specifically targeted challenging examples of casework such as aging or masked faces. The likelihood ratios we calculate are a function of the conditioning information provided by the test response dataset, and all likelihood ratios depend on the reference database. We believe that our likelihood ratios will apply to that aspect of casework that is similar in image quality and difficulty to the tests used in the studies we analyzed. Because of the large variations in recording devices, lighting, and capture settings, there may be no one “casework” to approximate. However, to the degree to which the black box studies and proficiency tests approximate casework, our likelihood ratios are a good estimate of what might be found in actual casework or at least that portion of casework where an examiner is sitting down to do a deliberate comparison (after easy exclusions have been pruned out).

## 7 IMPLICATION FOR CASEWORK AND CRIMINAL JUSTICE PARTNERS

The main conclusion of this article is that the likelihood ratios we observe are modest relative to the verbal statements of published articulation scales, as well as any given numerical LR equivalency of the verbal statements (see Tables 1 and 2). Organizations have been using different articulation scales with no scientific support, in terms of published validation or calibration studies, for the terms, and little acknowledgment of the problems associated with using uncalibrated or miscalibrated evaluative statements. Part of the issue may be that likelihood ratios were first developed for DNA, and the nature of DNA has produced extremely high likelihood ratios which, in our view, have influenced the creation of verbal equivalency scales. We view the disconnect between our likelihood ratio values and the associated verbal equivalency statements as an opportunity to revisit verbal equivalency scales. For example, when discussing the related concept of Bayes Factor, Lee and Wagenmakers [29] associated a range of values of 30–100 with the phrase “Very strong evidence in favor of H1,” which is much lower than verbal equivalency scales in forensics. Similarly, the UK Forensic Science Regulator's [30] guidance on evaluative opinions equates likelihood ratio values of 100–1000 as providing support that is ‘much more probable’ for one proposition over the alternative and suggests it would be unlikely that values above 1000 would be achieved if the available data is limited [30]. It is critical to remember that the likelihood ratio of a single piece of evidence is only one part of a wider case. A likelihood ratio cannot be considered by itself as a conclusion, and even a large likelihood ratio runs the risk of being overwhelmed by very small prior odds. This is similar to a situation where strong forensic evidence is overwhelmed by a very strong alibi.

Our likelihood ratios could help forensic scientists calibrate their verbal descriptions and improve the interpretation of the forensic evidence by fact finders, but fact finders must also understand the relation of the forensic evidence to the rest of the case. However, it is also important to acknowledge that our likelihood ratios are conditional on the examiners and image pairs from each study. For example, in all of the studies we used, the images depicted adult faces; therefore, our likelihood ratios may not be applicable to images of juveniles. Additionally, all of these comparisons are inherently subjective, as forensic facial examiners in real cases base their opinions on their own knowledge, experience, and beliefs. As a result, examiners can be swayed by conditioning information by which they determine the relative strength of support. This subjectivity highlights the role of human decision‐making in forensic facial comparison. Importantly, miscalibration is a source of bias. Even when examiners follow standardized procedures, their interpretations are influenced by several factors such as individual thresholds, prior experience, expectations about case context, and others [31, 32]. Such factors can lead to variation across examiners, with some adopting more conservative decision criteria and others expressing stronger conclusions for comparable evidence. These human elements likely contribute to the tendency we observed for verbal statements to overstate the corresponding likelihood ratios. Recognizing these influences is important for improving both examiner training and the communication of evidential strength [32]. Another potential limitation of our likelihood ratios is that examiners may become more risk‐averse when they know they are being tested. However, in the case of ordered probit models, such shifts in responding are easily accommodated by shifts in the locations of decision thresholds, which will leave the likelihood ratios unchanged.

We view the disconnect between the computed likelihood ratios and the proportion of examiners whose verbal statements overstate the LR (as highlighted in Table 2) as a serious concern. Even when verbal statements are well calibrated to a numerical strength of evidence, they are prone to misinterpretation by lay persons [33]. If these verbal statements are poorly calibrated to the intended numerical strength of evidence, the potential for confusion is even greater. Based on our findings, even if a fact finder relies on the numerical equivalencies given to the verbal statements of a forensic face examiner, their conclusion will most likely overinterpret the strength of the evidence. A key strength of this study is the breadth of data analyzed: across multiple error rate and proficiency tests, we consistently observe low LRs relative to the language typically used to convey determinations by facial examiners. Each study will have limitations (for example, some proficiency tests do not verify that all participants are facial examiners; it is difficult to know what “casework” represents in order to randomly sample from it). However, the studies under consideration represent the best summary of overall facial examinations currently available and therefore our best estimates of the behavior of facial comparison experts. It is not our intent to advocate for likelihood ratios to be presented in court directly, at least not without careful consideration, because laypersons struggle with likelihood ratios. Instead, we intend the present work to provoke dialogue within the forensic community about how best to convey the strength of support in examiner determinations so that these determinations more accurately reflect the strength of the evidence.

## CONFLICT OF INTEREST STATEMENT

The authors have no competing interests to declare.

## Supporting information


Appendix S1.

## Data Availability

The analyses generated for this study are available on Open Science Framework on the following link: https://osf.io/j2w49/?view_only=e9eaa514f96249a0b045fc22bd43eb68.
