# Supplementary material for: Do evaluative statements in facial identification overstate the strength of the evidence?
Source: J Forensic Sci. 2026 Jan 26;71(2):799–810. doi: 10.1111/1556-4029.70265 (PMC12967692; doi:10.1111/1556-4029.70265)
Supplement: Supplementary file 1 — Appendix S1. [file JFO-71-799-s001.docx]

# **Supplementary Information**

In this document we provide additional details about the Ordered Probit Likelihood Ratio model, along with the results of several sensitivity analyses that explore different assumptions and distributions for the ordered probit model.

## **Ordered Probit Model Description**

The ordered probit model summarizes the distribution of verbal responses from examiners in a black box study by assuming these verbal responses are an expression of a continuous value along a latent axis. Using a normal distribution, it estimates the location of a normal distribution and a set of decision thresholds along the latent axis that leads to the observed collection of responses.

Taking facial identification as an example, the ordered probit model summarizes distributions of examiner responses for each comparison to estimate a normal distribution along a latent axis where the right endpoint is the most support imaginable for the same source proposition and the left endpoint is the least support imaginable for the same source proposition. We assume that at the conclusion of each comparison, each examiner mentally ends up with a value along this latent axis that represents the amount of support for the same source proposition (or the relative support for the same- and different-sources propositions). We then assume that the collection of values across examiners along the latent axis can be summarized with a normal distribution for each pair of faces in the database. The model then assumes that the location along the latent axis is translated by examiners into one of the five determinations (in a 5 point conclusion scale) through the application of four thresholds, such that if the latent value falls above the highest threshold the examiner produces a Strong Support for Common Source determination, and if the value falls below the lowest threshold the examiner produces an Exclusion determination. Values in between the outer two thresholds result in one of the three determinations as proscribed by the two interior thresholds. The ordered probit model predicts the response frequency for each determination based on the area under the normal distribution between different thresholds, which reflect the system-wide behavior and do not represent the thresholds of a single examiner.

We use MCMC procedures to determine the most credible parameters for the ordered probit model, which summarize the support for the same source proposition offered by each pair. The mean (µ) in the normal distribution corresponds to the typical level of support for the two propositions as indicated by the examiner responses, where higher µ values indicate greater support for the same source proposition. The standard deviation σ reflects the consistency among examiners. We obtain the most credible values of µ and σ for each pair, along with the interior thresholds. Once we obtain the distribution of µ and σ parameters for each pair along with the two estimated thresholds, we can use these in combination with the ground truth (common source or different source) and two other assumptions (described in a later section) to calculate ordered probit likelihood ratios for individual image pairs.

**Ordered Probit Model Assumptions**

Before applying the ordered probit model to individual datasets, we would like to address the assumptions that underlie the model. The ordered probit model is a generalization of Signal Detection Theory, which is one of the major theoretical frameworks of the last 60 years. A search of Google Scholar for the term “Signal Detection Theory” recovers over 120,000 papers and books on the topic. The concept of a latent variable space is a core assumption of both signal detection theory and the ordered probit model.

The primary assumption is the normal (Gaussian) distribution. It has a long history within science. It was originally proposed by Fechner (1860) and applied to engineering and human performance by Swets. It results from the central limit theorem, which says that as you combine samples from a parent distribution you get a normal distribution even if the parent distribution is not normally distributed. Many non-Gaussian distributions tend to give similar results [1].

The latent space is not infinitely flexible. The key insight here is that if you monotonically change the scale, you shift BOTH the mated and nonmated distributions. The likelihood ratio is the ratio of the heights of the mated and nonmated distributions at every location along the latent axis, and is therefore unaffected by distortions of the latent axis. You could drag the mated curve to the right by distorting the right side of the latent axis, but you would drag along the right tail of the nonmated curve with it, therefore leaving the likelihood ratios unchanged.

The lack of real units does not affect the interpretation of the scale; for example, Fahrenheit has rather arbitrary units of 32 and 212 for the freezing and boiling points of water at sea level, but we use temperature all the time just fine. These numbers can be converted to Celsius, and while the numbers may change, the decision to take a jacket out with you will not depend on which units you are using. You experience cold regardless of which scale you use. Likewise, any scale can be used for the latent axis without loss of generality due to the way that likelihood ratios are calculated and the robustness to monotonic changes discussed above.

The second major assumption is that there is a set of system-wide thresholds that can be applied to all examiners. This is certainly not accurate, but probably a reasonable approximation given that a detective will not know which facial examiner will perform a comparison.

The third assumption is one of independence between subjects and image pairs. This is more complex, because in each study each subject gave responses to each image pair. While each image pair is probably independent (the same person probably did not have more than one image pair in the study), the examiner’s responses are not. This may have the effect of reducing the estimated error rate [2] and therefore artificially increasing the computed likelihood ratios. Thus our likelihood ratios could be seen as a ceiling on true performance.

By assuming independence, we can add all the mated and nonmated distributions together to create a reference database that expresses the probability of observing each value along the latent axis given *any* mated or nonmated pair. The likelihood ratio is the ratio of these two probability distributions at every point along the latent axis

The final assumption is that the likelihood ratio for a given pair in the database is the likelihood ratio that corresponds to the mean of the normal distribution.

Together these four assumptions translate collections of responses for each item in an error rate study into a set of normal distributions, which, when combined with ground truth, create likelihood ratios for that image pair. These likelihood ratios are a function of the conditioning information for that error rate study, which will include the collection of images as well as the examiners who participated in the study. The likelihood ratios will apply to casework to the degree to which the error rate study corresponds to that casework.

**Proficiency Test Datasets**

In the sections below we describe the proficiency tests that we describe in the manuscript, along with the ordered probit model likelihood ratios.

**2021, 2022, 2023 I3 Proficiency Tests Data**

Table S1 presents data from the 2021, 2022, 2023 I3 Proficiency tests data [3]. The ordered probit likelihood ratios for same source pairs range from 4.04 to 264 and are associated with µ values ranging from 3.23 to 6.09. For different source pairs, the ordered probit likelihood ratios ranged from 0.0027 to 0.91 and were associated with µ values ranging from 0.21 to 2.91. The left side of Figure S1 shows the relative likelihood of observing a given latent value for each mated (light blue curves) or nonmated (light red curves) comparison for the 2021, 2022, 2023 I3 Proficiency tests data. The right side shows the likelihood ratio values for different values along the latent axis for the 2021, 2022, 2023 I3 Proficiency tests data.

TABLE S1 Data from the 2021, 2022, 2023 I3 Proficiency tests. We calculated the µ and σ value using the ordered probit model, and sorted the pairs from the lowest µ to the highest µ. The numbers on the right side of the table represent the number of examiners who responded with “Exclusion”, “Support for Exclusion”, “Inconclusive”, “Support for Common Source”, “Strong Support for Common Source”. Each pair's ground truth is indicated by the column “Mated” with False referring to nonmated pairs and True referring to mated pairs.

| **pairID** | **Mated** | **μ** | **σ** | **Likelihood Ratio** | **Exclusion** | **Support for Exclusion** | **Inconclusive** | **Support for Common Source** | **Strong Support for Common Source** |
| --- | --- | --- | --- | --- | --- | --- | --- | --- | --- |
| **7_2021** | FALSE | 0.21 | 1.18 | 0.00 | 37 | 4 | 0 | 0 | 1 |
| **11_2023** | FALSE | 0.66 | 0.95 | 0.01 | 28 | 7 | 0 | 0 | 0 |
| **14_2021** | FALSE | 0.92 | 0.98 | 0.01 | 30 | 11 | 0 | 1 | 0 |
| **6_2021** | FALSE | 0.93 | 0.90 | 0.01 | 30 | 12 | 0 | 0 | 0 |
| **13_2022** | FALSE | 1.04 | 0.98 | 0.01 | 21 | 8 | 2 | 0 | 0 |
| **15_2022** | FALSE | 1.05 | 1.06 | 0.01 | 21 | 8 | 0 | 2 | 0 |
| **17_2023** | FALSE | 1.06 | 0.89 | 0.01 | 23 | 12 | 0 | 0 | 0 |
| **2_2021** | FALSE | 1.15 | 1.08 | 0.02 | 27 | 11 | 1 | 3 | 0 |
| **6_2022** | FALSE | 1.22 | 0.87 | 0.02 | 18 | 13 | 0 | 0 | 0 |
| **18_2021** | FALSE | 1.29 | 0.92 | 0.02 | 24 | 15 | 3 | 0 | 0 |
| **1_2023** | FALSE | 1.32 | 0.92 | 0.03 | 19 | 15 | 0 | 1 | 0 |
| **18_2022** | FALSE | 1.36 | 1.09 | 0.03 | 17 | 12 | 0 | 1 | 1 |
| **2_2022** | FALSE | 1.40 | 0.94 | 0.03 | 16 | 13 | 1 | 1 | 0 |
| **4_2023** | FALSE | 1.42 | 0.95 | 0.03 | 18 | 14 | 2 | 1 | 0 |
| **12_2022** | FALSE | 1.42 | 0.86 | 0.03 | 15 | 15 | 1 | 0 | 0 |
| **12_2021** | FALSE | 1.43 | 0.99 | 0.03 | 22 | 15 | 3 | 2 | 0 |
| **10_2022** | FALSE | 1.53 | 0.90 | 0.04 | 14 | 14 | 3 | 0 | 0 |
| **10_2023** | FALSE | 1.55 | 0.93 | 0.04 | 16 | 15 | 3 | 1 | 0 |
| **17_2022** | FALSE | 1.66 | 0.88 | 0.05 | 12 | 15 | 4 | 0 | 0 |
| **9_2021** | FALSE | 1.66 | 1.02 | 0.05 | 18 | 18 | 1 | 5 | 0 |
| **8_2023** | FALSE | 1.72 | 0.96 | 0.06 | 12 | 21 | 0 | 1 | 1 |
| **5_2021** | FALSE | 1.72 | 1.04 | 0.06 | 18 | 15 | 4 | 5 | 0 |
| **5_2022** | FALSE | 1.75 | 0.96 | 0.07 | 12 | 13 | 4 | 2 | 0 |
| **9_2022** | FALSE | 1.76 | 1.02 | 0.07 | 14 | 5 | 12 | 0 | 0 |
| **3_2021** | FALSE | 1.79 | 1.09 | 0.07 | 17 | 16 | 4 | 4 | 1 |
| **3_2023** | FALSE | 2.18 | 0.96 | 0.18 | 8 | 15 | 6 | 6 | 0 |
| **14_2023** | FALSE | 2.25 | 1.06 | 0.21 | 9 | 12 | 7 | 6 | 1 |
| **15_2021** | FALSE | 2.28 | 1.06 | 0.22 | 10 | 16 | 6 | 9 | 1 |
| **19_2023** | FALSE | 2.32 | 1.11 | 0.25 | 8 | 15 | 3 | 7 | 2 |
| **19_2021** | FALSE | 2.57 | 0.96 | 0.43 | 5 | 14 | 15 | 6 | 2 |
| **12_2023** | FALSE | 2.91 | 1.09 | 0.91 | 4 | 10 | 5 | 13 | 3 |
| **17_2021** | TRUE | 3.23 | 1.11 | 1.82 | 4 | 8 | 4 | 21 | 5 |
| **8_2021** | TRUE | 3.25 | 0.76 | 1.91 | 1 | 1 | 17 | 22 | 1 |
| **18_2023** | TRUE | 3.45 | 0.96 | 2.90 | 2 | 3 | 4 | 23 | 3 |
| **20_2023** | TRUE | 3.62 | 0.90 | 4.07 | 1 | 1 | 6 | 23 | 4 |
| **8_2022** | TRUE | 3.81 | 0.96 | 6.02 | 1 | 1 | 3 | 20 | 6 |
| **20_2022** | TRUE | 3.82 | 1.05 | 6.17 | 0 | 4 | 5 | 13 | 9 |
| **1_2022** | TRUE | 3.84 | 0.95 | 6.47 | 1 | 1 | 2 | 21 | 6 |
| **16_2023** | TRUE | 3.85 | 0.96 | 6.60 | 0 | 4 | 2 | 21 | 8 |
| **4_2022** | TRUE | 4.27 | 0.94 | 15.36 | 0 | 0 | 3 | 16 | 12 |
| **1_2021** | TRUE | 4.29 | 0.93 | 15.85 | 0 | 2 | 0 | 24 | 16 |
| **19_2022** | TRUE | 4.30 | 0.98 | 16.04 | 0 | 0 | 4 | 14 | 13 |
| **5_2023** | TRUE | 4.33 | 1.04 | 17.18 | 1 | 0 | 2 | 17 | 15 |
| **16_2022** | TRUE | 4.35 | 0.88 | 17.67 | 0 | 0 | 1 | 18 | 12 |
| **11_2021** | TRUE | 4.38 | 1.12 | 18.87 | 1 | 2 | 2 | 17 | 20 |
| **7_2022** | TRUE | 4.40 | 1.15 | 19.60 | 0 | 4 | 1 | 10 | 16 |
| **16_2021** | TRUE | 4.47 | 0.98 | 22.68 | 0 | 2 | 0 | 20 | 20 |
| **2_2023** | TRUE | 4.56 | 0.96 | 26.78 | 0 | 0 | 2 | 15 | 18 |
| **7_2023** | TRUE | 4.58 | 0.96 | 27.90 | 0 | 1 | 0 | 16 | 18 |
| **13_2023** | TRUE | 4.61 | 0.88 | 29.67 | 0 | 0 | 0 | 17 | 18 |
| **10_2021** | TRUE | 4.63 | 0.99 | 30.75 | 0 | 1 | 1 | 17 | 23 |
| **11_2022** | TRUE | 4.68 | 0.90 | 34.10 | 0 | 0 | 0 | 14 | 17 |
| **13_2021** | TRUE | 5.06 | 0.99 | 70.80 | 0 | 0 | 1 | 11 | 30 |
| **4_2021** | TRUE | 5.13 | 1.00 | 81.23 | 0 | 0 | 1 | 10 | 31 |
| **6_2023** | TRUE | 5.24 | 0.96 | 100.16 | 0 | 0 | 0 | 8 | 27 |
| **20_2021** | TRUE | 5.30 | 1.06 | 112.61 | 0 | 1 | 0 | 8 | 33 |
| **3_2022** | TRUE | 5.37 | 0.98 | 126.68 | 0 | 0 | 0 | 6 | 25 |
| **15_2023** | TRUE | 5.45 | 0.98 | 147.17 | 0 | 0 | 0 | 6 | 29 |
| **14_2022** | TRUE | 5.49 | 0.98 | 160.10 | 0 | 0 | 0 | 5 | 26 |
| **9_2023** | TRUE | 6.09 | 1.00 | 506.44 | 0 | 0 | 0 | 2 | 33 |
|  |  |  |  |  |  |  |  |  |  |

FIGURE S1 Left panel: Relative likelihood of observing a given latent value for each mated (light blue curves) or nonmated (light red curves) comparison for the 2021, 2022, 2023 I3 Proficiency tests data. The parameters for each normal distribution were derived from the ordered probit model fit to all eleven conclusions for each comparison. The thick red curve corresponds to the sum of light red curves. It represents the relative likelihood of observing any nonmated comparison at each value of the latent axis. The thick blue curve represents the relative likelihood of observing any mated comparison at each value of the latent axis. The vertical lines correspond to the median threshold values that divide the latent axis to produce the estimate of the proportion of responses in each bin.

Right panel: Likelihood ratio values for different values along the latent axis for the 2021, 2022, 2023 I3 Proficiency tests data. The y axis is plotted on a log(10) axis. Likelihood ratios for individual mated pairs are shown as blue circles, and likelihood ratios for nonmated pairs are shown as red circles.

**2018 and 2022 ENFSI Examiner Data**

The left side of Figure S2 shows the relative likelihood of observing a given latent value for each mated (light blue curves) or nonmated (light red curves) comparison for the 2018 and 2022 ENFSI data. The right side shows the likelihood ratio values for different values along the latent axis for the 2018 and 2022 ENFSI data.

|  |  |
| --- | --- |
|  |  |

FIGURE S2 **Left panel**: Relative likelihood of observing a given latent value for each mated (light blue curves) or nonmated (light red curves) comparison for the 2018 and 2022 ENFSI Examiner data. The parameters for each normal distribution were derived from the ordered probit model fit to all eleven conclusions for each comparison. The thick red curve corresponds to the sum of light red curves. It represents the relative likelihood of observing any nonmated comparison at each value of the latent axis. The thick blue curve represents the relative likelihood of observing any mated comparison at each value of the latent axis while the thick red curve represents the relative likelihood of observing any nonmated comparison at each value of the latent axis. The vertical lines correspond to the median threshold values that divide the latent axis to produce the estimate of the proportion of responses in each bin. P-labels correspond to positive support statements and m-labels correspond to negative support statements (support for different persons).

**Right panel**: Likelihood ratio values for different values along the latent axis for the 2018 *and 2022* ENFSI *Examiner* data. This curve is the ratio of the thick blue divided by the thick red curve in the left panel. The y axis is plotted on a log(10) axis. Likelihood ratios for individual mated pairs are shown as blue circles, and likelihood ratios for nonmated pairs are shown as red circles.

|  |  |
| --- | --- |

## **Teams Data**

The tables (S2 to S4) and figures (S3 to S5) shown below refer to the analysis of the Teams data from the 2018 and 2022 ENFSI proficiency tests.

TABLE S2 Data from the 2018 and 2022 European Network of Forensic Science Institutes investigation for Teams. PairIDs starting with “Q” with corresponds to the 2018 ENFSI data while pairIDs starting with “Trial” corresponds to the 2022 ENFSI data. We calculated the µ and σ value using the ordered probit model, and sorted the pairs from the lowest µ to the highest µ. The numbers on the right side of the table represent the number of examiners who responded with “Extremely Strong Support for Different People” (m5) , “Very Strong Support for Different People” (m4), “Strong Support for Different People” (m3), “Support for Different People” (m2), and “Weak Support for Different People” (m1), “Inconclusive” (zero), “Weak Support for Same Person” (p1), “Support for Same Person” (p2), “Strong Support for Same Person” (p3), “Very Strong Support for Same Person” (p4), and “Extremely Strong Support for Same Person” (p5). Each pair's ground truth is indicated by the column “Mated” with False referring to nonmated pairs and True referring to mated pairs.

| **pairID** | **Mated** | **mu** | **sigma** | **LR** | **Proportion Examiners Choosing a Higher Verbal Equivalency Than The LR** | **m5** | **m4** | **m3** | **m2** | **m1** | **zero** | **p1** | **p2** | **p3** | **p4** | **p5** |
| --- | --- | --- | --- | --- | --- | --- | --- | --- | --- | --- | --- | --- | --- | --- | --- | --- |
| **Q3-nm** | FALSE | 2.10 | 1.63 | 0.01 |  | 7 | 4 | 6 | 1 | 0 | 0 | 1 | 0 | 0 | 0 | 0 |
| **Q4-nm** | FALSE | 2.56 | 1.56 | 0.02 |  | 4 | 6 | 6 | 2 | 0 | 0 | 0 | 1 | 0 | 0 | 0 |
| **Q2-nm** | FALSE | 3.04 | 1.30 | 0.03 |  | 2 | 4 | 7 | 5 | 0 | 1 | 0 | 0 | 0 | 0 | 0 |
| **Trial 10** | FALSE | 3.38 | 1.28 | 0.05 |  | 1 | 5 | 7 | 7 | 2 | 0 | 1 | 0 | 0 | 0 | 0 |
| **Q6-nm** | FALSE | 3.49 | 1.80 | 0.06 |  | 3 | 5 | 1 | 4 | 1 | 3 | 1 | 1 | 0 | 0 | 0 |
| **Trial 1** | FALSE | 3.92 | 1.54 | 0.09 |  | 2 | 2 | 6 | 3 | 6 | 1 | 3 | 0 | 0 | 0 | 0 |
| **Q14-nm** | FALSE | 4.17 | 1.64 | 0.12 |  | 1 | 1 | 7 | 3 | 3 | 0 | 1 | 3 | 0 | 0 | 0 |
| **Q9-nm** | FALSE | 4.18 | 1.48 | 0.12 |  | 0 | 3 | 6 | 0 | 5 | 3 | 1 | 1 | 0 | 0 | 0 |
| **Trial 13** | FALSE | 4.23 | 1.28 | 0.13 |  | 0 | 2 | 5 | 6 | 7 | 1 | 1 | 1 | 0 | 0 | 0 |
| **Trial 4** | FALSE | 4.30 | 1.20 | 0.14 |  | 0 | 2 | 2 | 11 | 3 | 3 | 2 | 0 | 0 | 0 | 0 |
| **Trial 9** | FALSE | 4.41 | 1.17 | 0.16 |  | 0 | 2 | 4 | 1 | 12 | 4 | 0 | 0 | 0 | 0 | 0 |
| **Trial 18** | FALSE | 4.71 | 1.34 | 0.23 |  | 0 | 2 | 1 | 6 | 8 | 2 | 2 | 2 | 0 | 0 | 0 |
| **Trial 11** | FALSE | 4.91 | 1.68 | 0.30 |  | 0 | 1 | 5 | 6 | 3 | 1 | 3 | 1 | 3 | 0 | 0 |
| **Trial 17** | FALSE | 4.92 | 1.45 | 0.30 |  | 0 | 0 | 4 | 5 | 9 | 0 | 0 | 4 | 1 | 0 | 0 |
| **Q13-nm** | FALSE | 4.97 | 1.37 | 0.32 |  | 0 | 0 | 4 | 4 | 0 | 7 | 2 | 2 | 0 | 0 | 0 |
| **Trial 3** | FALSE | 4.98 | 1.39 | 0.32 |  | 0 | 1 | 1 | 6 | 6 | 6 | 1 | 0 | 2 | 0 | 0 |
| **Trial 7** | FALSE | 5.16 | 1.57 | 0.40 |  | 0 | 0 | 3 | 5 | 8 | 2 | 0 | 3 | 1 | 1 | 0 |
| **Trial 14** | TRUE | 5.81 | 1.14 | 0.85 |  | 0 | 1 | 0 | 0 | 2 | 8 | 9 | 3 | 0 | 0 | 0 |
| **Trial 12** | TRUE | 6.23 | 0.95 | 1.37 | 0.65 | 0 | 0 | 0 | 0 | 1 | 7 | 9 | 5 | 1 | 0 | 0 |
| **Trial 15** | TRUE | 6.27 | 1.15 | 1.42 | 0.65 | 0 | 0 | 0 | 0 | 3 | 5 | 8 | 6 | 0 | 1 | 0 |
| **Trial 6** | TRUE | 6.38 | 1.68 | 1.61 | 0.65 | 0 | 1 | 1 | 0 | 1 | 5 | 6 | 5 | 3 | 0 | 1 |
| **Q15-m** | TRUE | 6.43 | 1.92 | 1.70 | 0.58 | 0 | 0 | 2 | 4 | 2 | 0 | 0 | 3 | 6 | 2 | 0 |
| **Trial 5** | TRUE | 6.80 | 1.53 | 2.60 | 0.48 | 0 | 1 | 0 | 0 | 1 | 2 | 8 | 3 | 7 | 1 | 0 |
| **Q16-m** | TRUE | 6.88 | 1.56 | 2.87 | 0.63 | 0 | 0 | 1 | 1 | 0 | 2 | 3 | 7 | 3 | 2 | 0 |
| **Q12-m** | TRUE | 7.12 | 1.37 | 3.83 | 0.68 | 0 | 0 | 0 | 1 | 0 | 1 | 4 | 8 | 3 | 2 | 0 |
| **Q11-m** | TRUE | 7.38 | 1.28 | 5.32 | 0.74 | 0 | 0 | 0 | 0 | 1 | 1 | 3 | 5 | 8 | 1 | 0 |
| **Trial 8** | TRUE | 7.42 | 1.52 | 5.60 | 0.78 | 0 | 1 | 0 | 0 | 0 | 0 | 4 | 8 | 7 | 3 | 0 |
| **Q10-m** | TRUE | 7.98 | 1.60 | 12.19 | 0.53 | 0 | 0 | 0 | 1 | 0 | 0 | 2 | 6 | 4 | 5 | 1 |
| **Q20-m** | TRUE | 8.25 | 1.71 | 18.22 | 0.63 | 0 | 0 | 0 | 1 | 0 | 2 | 0 | 4 | 3 | 8 | 1 |
| **Q19-m** | TRUE | 8.43 | 1.30 | 24.14 | 0.74 | 0 | 0 | 0 | 0 | 0 | 0 | 1 | 4 | 8 | 5 | 1 |
| **Q7-m** | TRUE | 8.50 | 1.75 | 27.13 | 0.58 | 0 | 0 | 0 | 0 | 0 | 2 | 1 | 5 | 2 | 6 | 3 |
| **Trial 19** | TRUE | 8.62 | 1.92 | 32.42 | 0.74 | 0 | 0 | 1 | 0 | 1 | 0 | 2 | 2 | 6 | 7 | 4 |
| **Trial 20** | TRUE | 8.85 | 1.65 | 47.57 | 0.7 | 0 | 0 | 0 | 0 | 1 | 0 | 1 | 5 | 2 | 11 | 3 |
| **Q1-m** | TRUE | 9.26 | 1.21 | 92.20 | 0.95 | 0 | 0 | 0 | 0 | 0 | 0 | 0 | 1 | 6 | 10 | 2 |
| **Trial 2** | TRUE | 9.31 | 1.49 | 101.04 | 0.61 | 0 | 0 | 0 | 0 | 0 | 0 | 0 | 4 | 5 | 9 | 5 |
| **Q8-m** | TRUE | 9.39 | 1.12 | 114.65 | 0.68 | 0 | 0 | 0 | 0 | 0 | 0 | 0 | 0 | 6 | 11 | 2 |
| **Q18-m** | TRUE | 9.43 | 1.27 | 122.72 | 0.68 | 0 | 0 | 0 | 0 | 0 | 0 | 0 | 1 | 5 | 10 | 3 |
| **Q5-m** | TRUE | 9.61 | 1.32 | 165.39 | 0.74 | 0 | 0 | 0 | 0 | 0 | 0 | 0 | 1 | 4 | 10 | 4 |
| **Q17-m** | TRUE | 9.69 | 1.50 | 188.14 | 0.63 | 0 | 0 | 0 | 0 | 0 | 0 | 0 | 1 | 6 | 6 | 6 |
| **Trial 16** | TRUE | 10.82 | 1.70 | 1164.43 | 0.83 | 0 | 0 | 0 | 0 | 0 | 0 | 0 | 1 | 3 | 5 | 14 |

|  |  |
| --- | --- |

|  |  |
| --- | --- |
|  |  |

FIGURE S3 **Left panel**: Relative likelihood of observing a given latent value for each mated (light blue curves) or nonmated (light red curves) comparison for the 2018 and 2022 ENFSI Team data. The parameters for each normal distribution were derived from the ordered probit model fit to all eleven conclusions for each comparison. The thick red curve corresponds to the sum of light red curves. It represents the relative likelihood of observing any nonmated comparison at each value of the latent axis. The thick blue curve represents the relative likelihood of observing any mated comparison at each value of the latent axis.

**Right panel**: Likelihood ratio values for different values along the latent axis for the 2018 *and 2022* ENFSI Team data. The y axis is plotted on a log(10) axis. The log of the likelihood ratio can be observed directly as the difference between the thick blue and thick red curves in the top. The blue region illustrates the approximate range of image pairs with majority ID decisions.

TABLE S3 Data from the 2021 European Network of Forensic Science Institutes investigation for Teams. We calculated the µ and σ value using the ordered probit model, and sorted the pairs from the lowest µ to the highest µ. The numbers on the right side of the table represent the number of examiners who responded with “Extremely Strong Support for Different People” (m5) , “Very Strong Support for Different People” (m4), “Strong Support for Different People” (m3), “Support for Different People” (m2), and “Weak Support for Different People” (m1), “Inconclusive” (zero), “Weak Support for Same Person” (p1), “Support for Same Person” (p2), “Strong Support for Same Person” (p3), “Very Strong Support for Same Person” (p4), and “Extremely Strong Support for Same Person” (p5). Each pair's ground truth is indicated by the column “Mated” with False referring to nonmated pairs and True referring to mated pairs.

| **pairID** | **Mated** | **mu** | **sigma** | **LR** | **Proportion Examiners Choosing a Higher Verbal Equivalency Than The LR** | **m5** | **m4** | **m3** | **m2** | **m1** | **zero** | **p1** | **p2** | **p3** | **p4** | **p5** |
| --- | --- | --- | --- | --- | --- | --- | --- | --- | --- | --- | --- | --- | --- | --- | --- | --- |
| **C14NM** | FALSE | 2.45 | 1.36 | 0.01 |  | 9 | 12 | 12 | 3 | 3 | 0 | 0 | 0 | 0 | 0 | 0 |
| **C13NM** | FALSE | 2.85 | 1.41 | 0.02 |  | 7 | 10 | 10 | 7 | 5 | 0 | 0 | 0 | 0 | 0 | 0 |
| **C18NM** | FALSE | 3.49 | 1.35 | 0.05 |  | 3 | 6 | 13 | 8 | 8 | 0 | 1 | 0 | 0 | 0 | 0 |
| **C4NM** | FALSE | 3.71 | 1.63 | 0.07 |  | 3 | 5 | 13 | 8 | 7 | 2 | 0 | 0 | 0 | 0 | 1 |
| **C11NM** | FALSE | 3.78 | 1.25 | 0.08 |  | 1 | 6 | 11 | 9 | 10 | 2 | 0 | 0 | 0 | 0 | 0 |
| **C20NM** | FALSE | 3.85 | 1.54 | 0.08 |  | 2 | 6 | 10 | 10 | 7 | 2 | 1 | 0 | 0 | 1 | 0 |
| **C5NM** | FALSE | 4.63 | 1.81 | 0.23 |  | 1 | 4 | 9 | 7 | 8 | 3 | 3 | 0 | 3 | 0 | 1 |
| **C8NM** | FALSE | 4.84 | 1.40 | 0.29 |  | 0 | 2 | 6 | 8 | 9 | 11 | 2 | 0 | 0 | 1 | 0 |
| **C16NM** | FALSE | 5.44 | 1.19 | 0.59 |  | 0 | 0 | 4 | 1 | 13 | 15 | 4 | 1 | 1 | 0 | 0 |
| **C3M** | TRUE | 5.76 | 1.36 | 0.85 |  | 0 | 0 | 2 | 6 | 5 | 15 | 7 | 1 | 2 | 1 | 0 |
| **C15NM** | FALSE | 6.65 | 1.30 | 2.16 | 0.33 | 0 | 0 | 0 | 3 | 0 | 14 | 9 | 8 | 3 | 2 | 0 |
| **C6M** | TRUE | 6.94 | 1.18 | 2.87 | 0.49 | 0 | 0 | 0 | 2 | 1 | 5 | 12 | 15 | 3 | 1 | 0 |
| **C12M** | TRUE | 7.08 | 1.57 | 3.27 | 0.54 | 0 | 0 | 2 | 2 | 2 | 4 | 8 | 9 | 8 | 4 | 0 |
| **C2M** | TRUE | 7.39 | 1.28 | 4.37 | 0.64 | 0 | 0 | 0 | 2 | 0 | 4 | 8 | 12 | 11 | 2 | 0 |
| **C1M** | TRUE | 7.46 | 1.40 | 4.67 | 0.62 | 0 | 0 | 1 | 0 | 1 | 6 | 7 | 8 | 12 | 4 | 0 |
| **C19M** | TRUE | 7.54 | 1.25 | 5.02 | 0.64 | 0 | 0 | 0 | 1 | 0 | 3 | 10 | 12 | 9 | 4 | 0 |
| **C17M** | TRUE | 7.77 | 1.25 | 6.20 | 0.74 | 0 | 0 | 0 | 1 | 1 | 1 | 7 | 9 | 17 | 3 | 0 |
| **C7M** | TRUE | 8.73 | 1.43 | 15.66 | 0.72 | 0 | 0 | 1 | 0 | 0 | 0 | 2 | 8 | 10 | 16 | 2 |
| **C10M** | TRUE | 9.44 | 1.26 | 34.25 | 0.92 | 0 | 0 | 0 | 0 | 0 | 0 | 1 | 2 | 13 | 16 | 7 |
| **C9M** | TRUE | 9.53 | 1.38 | 37.75 | 0.9 | 0 | 0 | 0 | 0 | 0 | 0 | 1 | 3 | 13 | 12 | 10 |

|  |  |
| --- | --- |

|  |  |
| --- | --- |
|  |  |

FIGURE S4 **Left panel**: Relative likelihood of observing a given latent value for each mated (light blue curves) or nonmated (light red curves) comparison for the 2021 ENFSI Examiner data. The parameters for each normal distribution were derived from the ordered probit model fit to all eleven conclusions for each comparison. The thick red curve corresponds to the sum of light red curves. It represents the relative likelihood of observing any nonmated comparison at each value of the latent axis. The thick blue curve represents the relative likelihood of observing any mated comparison at each value of the latent axis.

**Right panel**: Likelihood ratio values for different values along the latent axis for the 2021 ENFSI Examiner data. The y axis is plotted on a log(10) axis. The log of the likelihood ratio can be observed directly as the difference between the thick blue and thick red curves in the top. The blue region illustrates the approximate range of image pairs with majority ID decisions.

TABLE S4 Data from the 2021 European Network of Forensic Science Institutes investigation for Examiners. We calculated the µ and σ value using the ordered probit model, and sorted the pairs from the lowest µ to the highest µ. The numbers on the right side of the table represent the number of examiners who responded with “Extremely Strong Support for Different People” (m5) , “Very Strong Support for Different People” (m4), “Strong Support for Different People” (m3), “Support for Different People” (m2), and “Weak Support for Different People” (m1), “Inconclusive” (zero), “Weak Support for Same Person” (p1), “Support for Same Person” (p2), “Strong Support for Same Person” (p3), “Very Strong Support for Same Person” (p4), and “Extremely Strong Support for Same Person” (p5). Each pair's ground truth is indicated by the column “Mated” with False referring to nonmated pairs and True referring to mated pairs.

| **pairID** | **Mated** | **mu** | **sigma** | **LR** | **Proportion Examiners Choosing a Higher Verbal Equivalency Than The LR for LR>1** | **m5** | **m4** | **m3** | **m2** | **m1** | **zero** | **p1** | **p2** | **p3** | **p4** | **p5** |
| --- | --- | --- | --- | --- | --- | --- | --- | --- | --- | --- | --- | --- | --- | --- | --- | --- |
| **C14NM** | FALSE | 2.27 | 1.71 | 0.10 |  | 19 | 16 | 14 | 8 | 2 | 0 | 0 | 2 | 0 | 0 | 0 |
| **C13NM** | FALSE | 2.57 | 1.41 | 0.12 |  | 14 | 15 | 16 | 12 | 4 | 0 | 0 | 0 | 0 | 0 | 0 |
| **C18NM** | FALSE | 3.29 | 1.33 | 0.19 |  | 6 | 12 | 13 | 20 | 7 | 3 | 0 | 0 | 0 | 0 | 0 |
| **C11NM** | FALSE | 3.33 | 1.41 | 0.20 |  | 5 | 13 | 18 | 13 | 3 | 8 | 1 | 0 | 0 | 0 | 0 |
| **C4NM** | FALSE | 3.42 | 1.93 | 0.21 |  | 8 | 11 | 19 | 8 | 7 | 1 | 1 | 3 | 3 | 0 | 0 |
| **C20NM** | FALSE | 3.84 | 1.52 | 0.29 |  | 4 | 10 | 8 | 17 | 12 | 5 | 3 | 2 | 0 | 0 | 0 |
| **C5NM** | FALSE | 3.90 | 1.83 | 0.30 |  | 3 | 13 | 14 | 10 | 8 | 2 | 5 | 3 | 3 | 0 | 0 |
| **C8NM** | FALSE | 4.64 | 1.17 | 0.52 |  | 0 | 3 | 8 | 13 | 14 | 15 | 6 | 2 | 0 | 0 | 0 |
| **C16NM** | FALSE | 5.06 | 1.33 | 0.72 |  | 1 | 0 | 8 | 6 | 16 | 13 | 10 | 6 | 1 | 0 | 0 |
| **C3M** | TRUE | 5.28 | 1.71 | 0.87 |  | 1 | 2 | 5 | 13 | 7 | 10 | 10 | 7 | 5 | 1 | 0 |
| **C12M** | TRUE | 5.63 | 2.35 | 1.16 | 0.49 | 3 | 3 | 6 | 10 | 3 | 6 | 8 | 9 | 10 | 0 | 3 |
| **C6M** | TRUE | 6.07 | 1.47 | 1.68 | 0.57 | 0 | 0 | 4 | 1 | 8 | 13 | 13 | 15 | 6 | 0 | 1 |
| **C2M** | TRUE | 6.56 | 1.85 | 2.50 | 0.57 | 1 | 0 | 3 | 6 | 2 | 5 | 9 | 18 | 14 | 2 | 1 |
| **C15NM** | FALSE | 6.59 | 1.66 | 2.57 | 0.52 | 0 | 1 | 1 | 2 | 4 | 12 | 9 | 18 | 11 | 1 | 2 |
| **C1M** | TRUE | 6.84 | 1.93 | 3.11 | 0.59 | 0 | 0 | 5 | 5 | 1 | 5 | 9 | 13 | 16 | 6 | 1 |
| **C17M** | TRUE | 7.24 | 1.65 | 4.20 | 0.67 | 0 | 0 | 1 | 2 | 3 | 1 | 13 | 20 | 11 | 9 | 1 |
| **C19M** | TRUE | 7.24 | 1.51 | 4.20 | 0.72 | 0 | 0 | 1 | 1 | 1 | 5 | 9 | 23 | 14 | 6 | 1 |
| **C10M** | TRUE | 8.28 | 2.19 | 8.16 | 0.79 | 0 | 1 | 1 | 3 | 1 | 2 | 5 | 7 | 17 | 16 | 8 |
| **C7M** | TRUE | 8.65 | 1.72 | 10.09 | 0.79 | 0 | 0 | 1 | 1 | 0 | 1 | 2 | 8 | 25 | 16 | 7 |
| **C9M** | TRUE | 9.01 | 1.94 | 12.42 | 0.8 | 0 | 1 | 1 | 0 | 0 | 0 | 2 | 8 | 18 | 20 | 11 |

| ** | ** |
| --- | --- |

FIGURE S5 **Left panel**: Relative likelihood of observing a given latent value for each mated (light blue curves) or nonmated (light red curves) comparison for the 2021 ENFSI Team data. The parameters for each normal distribution were derived from the ordered probit model fit to all eleven conclusions for each comparison. The thick red curve corresponds to the sum of light red curves. It represents the relative likelihood of observing any nonmated comparison at each value of the latent axis. The thick blue curve represents the relative likelihood of observing any mated comparison at each value of the latent axis.

**Right panel**: Likelihood ratio values for different values along the latent axis for the 2021 ENFSI Teams data. The y axis is plotted on a log(10) axis. The log of the likelihood ratio can be observed directly as the difference between the thick blue and thick red curves in the top. The blue region illustrates the approximate range of image pairs with majority ID decisions.

## **2024 CTS**

We computed likelihood ratios for the 2024 CTS investigation [4] which can be found in Table S5. The ordered probit likelihood ratios for same source pairs ranged from 1.82 to 506 and were associated with µ values ranging from 3.85 to 6.91. For different source pairs the ordered probit likelihood ratios ranged from 0.04 to 0.01 and were associated with µ values ranging from 2.23 to 1.46. The left side of Figure S6 shows the relative likelihood of observing a given latent value for each same source (light blue curves) or different source (light red curves) comparison and the right side shows the likelihood ratio values for different values along the latent axis for the 2024 CTS data.

TABLE S5 Data from the 2024 CTS investigation for Examiners. We calculated the µ and σ value using the ordered probit model, and sorted the pairs from the lowest µ to the highest µ. The numbers on the right side of the table represent the number of examiners who responded with “Strong Support for Different People” , “Support for Different People”, “Inconclusive”, “Support Common Source”, “Strong Support Common Source”. Each pair's ground truth is indicated by the column “Mated” with False referring to nonmated pairs and True referring to mated pairs.

| **pairID** | **Mated** | **μ** | **σ** | **Likelihood Ratio** | **strong_support_different** | **support_different** | **inconclusive** | **support_common** | **strong_support_**  **common** |
| --- | --- | --- | --- | --- | --- | --- | --- | --- | --- |
| **Q17** | FALSE | 1.46 | 1.36 | 0.01 | 33 | 19 | 7 | 4 | 0 |
| **Q3** | FALSE | 1.80 | 0.41 | 0.01 | 14 | 49 | 0 | 0 | 0 |
| **Q12** | FALSE | 1.81 | 0.41 | 0.02 | 13 | 50 | 0 | 0 | 0 |
| **Q10** | FALSE | 1.82 | 1.25 | 0.02 | 26 | 23 | 8 | 6 | 0 |
| **Q8** | FALSE | 1.91 | 0.58 | 0.02 | 14 | 48 | 0 | 1 | 0 |
| **Q5** | FALSE | 2.03 | 1.56 | 0.03 | 23 | 24 | 4 | 8 | 4 |
| **Q15** | FALSE | 2.09 | 1.36 | 0.03 | 24 | 12 | 22 | 4 | 1 |
| **Q1** | FALSE | 2.13 | 0.79 | 0.03 | 10 | 50 | 0 | 1 | 2 |
| **Q2** | FALSE | 2.14 | 0.70 | 0.03 | 9 | 51 | 0 | 2 | 1 |
| **Q13** | FALSE | 2.23 | 0.66 | 0.04 | 5 | 56 | 0 | 0 | 2 |
| **Q16** | TRUE | 3.85 | 0.95 | 4.04 | 3 | 0 | 11 | 29 | 10 |
| **Q11** | TRUE | 4.47 | 0.93 | 16.96 | 1 | 1 | 3 | 29 | 29 |
| **Q14** | TRUE | 4.68 | 0.70 | 24.65 | 0 | 1 | 0 | 25 | 37 |
| **Q7** | TRUE | 4.85 | 0.57 | 32.75 | 0 | 0 | 0 | 17 | 46 |
| **Q9** | TRUE | 5.01 | 1.20 | 40.61 | 1 | 1 | 1 | 19 | 41 |
| **Q4** | TRUE | 5.09 | 0.67 | 44.97 | 0 | 0 | 0 | 12 | 51 |
| **Q18** | TRUE | 5.79 | 1.21 | 82.48 | 0 | 1 | 0 | 8 | 54 |
| **Q6** | TRUE | 6.91 | 1.77 | 264.52 | 0 | 2 | 0 | 3 | 58 |

|  |
| --- |
|  |

FIGURE S6 **Left panel**: Relative likelihood of observing a given latent value for each mated (light blue curves) or nonmated (light red curves) comparison for the 2024 CTS data. The parameters for each normal distribution were derived from the ordered probit model fit to all five conclusions for each comparison. The thick red curve corresponds to the sum of light red curves. It represents the relative likelihood of observing any nonmated comparison at each value of the latent axis. The thick blue curve represents the relative likelihood of observing any mated comparison at each value of the latent axis. The vertical lines correspond to the median threshold values that divide the latent axis to produce the estimate of the proportion of responses in each bin.

**Right panel**: Likelihood ratio values for different values along the latent axis for the 202*4 CTS* data. The y axis is plotted on a log(10) axis. Likelihood ratios for individual mated pairs are shown as blue circles, and likelihood ratios for nonmated pairs are shown as red circles.

## **Sensitivity Analyses**

To the robustness of the Ordered Probit Model, we conducted various sensitivity analyses and examined the impact of variations in the model assumptions. The sensitivity analyses were done using the 2018 and 2022 European Network of Forensic Science Institutes investigation for Examiners as well as the NIST fingerprint examiners.

The first sensitivity analysis we looked at is the effect of turning off shrinkage. Making the Ordered Probit Model free to fit the data without any constraint of the standard deviation led to wider standard deviation in some cases (Figure S7 and S8). This results in complex changes in likelihood ratios, increasing some at the expense of decreasing others. This can be seen in Table S6 and S7 which shows wider standard deviation in some cases, and slight changes in likelihood ratios (both increase and decrease). Overall, the changes are modest and don’t suggest that this assumption significantly affect our computed likelihood ratios.

We also changed the priors by increasing the standard deviation on the prior for the μ values. The default model has a standard deviation equal to 3 * nLevels, where nLevels is the number of levels in the conclusion scale. We increased this to 10 * nLevels for the sensitivity analysis. This increase in the standard deviation of the priors on μ allowed the model to be less constrained by prior beliefs when estimating the μ parameters (Figure S9 and S10). This led to the likelihood ratio curve showing only a slight increase in the upper end showing that the wider mu priors don’t affect likelihood ratios.

Additionally, we ran the model using a t-distribution instead of a normal distribution. The t-distribution has heavier tails compared to the normal distribution. This means that extreme values are more likely under a t-distribution, leading to a higher probability of observing outliers or extreme events in the data (Figure S11 and S12).

None of these changes to the assumptions underlying the ordered probit model systematically increased the likelihood ratios across all values of μ. Overall these sensitivity analyses demonstrate that the modest likelihood ratios reported in the main paper are not a result of particular assumptions or choices of parameter values for prior distributions.

Finally, Figures S13 present direct comparisons across multiple versions of the ordered probit model, including tests using a broader range of prior values. None of these adjustments led to meaningful changes in the resulting likelihood ratios, largely because the size of the datasets rapidly dominates the influence of the priors.

**Turning off Shrinkage**

FIGURE S7 **Left panel**: Relative likelihood of observing a given latent value for each mated (light blue curves) or nonmated (light red curves) comparison for the NISTI data when turning off shrinkage. Shrinkage has been turned off making the model free to fit the data without any constraint, of the standard deviation leading to wider standard deviation in some cases.

**Right panel**: Likelihood ratio values for different values along the latent axis for the NIST data. The likelihood ratio values for different values along the latent axis exhibit a wider range without shrinkage. This is because the model is not being regularized towards a more constrained set of parameter values.

TABLE S6 Data from the Facial examiners from the NIST data when turning off shrinkage. We calculated the µ and σ value using the ordered probit model, and sorted the pairs from the lowest µ to the highest µ. The numbers on the right side of the table represent the number of examiners who responded with “strongly support that it is not the same person” (-3), “support that it is not the same person” (-2), and “support to some extent that it is not the same person” (-1), “Support neither that it is the same person nor that it is different persons ” (0), “Support to some extent that it is the same Person” (+1), “support for same person” (+2), “strong support for same Person” (+3). Each pair's ground truth is indicated by the column “Mated” with False referring to nonmated (different source) pairs and True referring to mated (same source) pairs. We observe wider standard deviation in some cases as well as complex changes in likelihood rations, increasing some while decreasing others.

| **pairID** | **Mated** | **mu** | **sigma** | **LR** | **-3** | **-2** | **-1** | **0** | **+1** | **+2** | **+3** |
| --- | --- | --- | --- | --- | --- | --- | --- | --- | --- | --- | --- |
| **04628d459_05252d168** | FALSE | 1.49 | 1.20 | 0.07 | 28 | 19 | 7 | 0 | 1 | 1 | 1 |
| **04509d590_05152d73** | FALSE | 1.92 | 1.20 | 0.10 | 19 | 24 | 9 | 0 | 0 | 4 | 1 |
| **05041d80_04986d102** | FALSE | 2.27 | 1.23 | 0.13 | 14 | 23 | 11 | 2 | 2 | 4 | 1 |
| **04605d295_05040d47** | FALSE | 2.64 | 1.41 | 0.20 | 11 | 20 | 13 | 0 | 4 | 8 | 1 |
| **04605d377_05017d229** | FALSE | 2.73 | 1.33 | 0.22 | 12 | 17 | 9 | 5 | 11 | 3 | 0 |
| **04884d76_05180d63** | FALSE | 2.87 | 0.79 | 0.27 | 4 | 19 | 19 | 13 | 1 | 1 | 0 |
| **05115d184_04778d77** | FALSE | 2.88 | 1.00 | 0.28 | 10 | 11 | 17 | 15 | 4 | 0 | 0 |
| **04670d374_04738d125** | FALSE | 2.98 | 1.10 | 0.32 | 6 | 17 | 16 | 4 | 11 | 3 | 0 |
| **04900d172_04900d155** | TRUE | 3.78 | 0.72 | 1.06 | 2 | 4 | 8 | 22 | 19 | 2 | 0 |
| **04937d81_04937d171** | TRUE | 3.99 | 1.08 | 1.42 | 4 | 4 | 9 | 8 | 25 | 5 | 2 |
| **05113d201_05113d21** | TRUE | 4.45 | 0.94 | 2.54 | 1 | 3 | 4 | 9 | 25 | 14 | 1 |
| **04379d484_04379d636** | TRUE | 4.57 | 1.14 | 2.91 | 1 | 6 | 6 | 2 | 22 | 18 | 2 |
| **05244d182_05244d77** | TRUE | 4.69 | 1.06 | 3.34 | 0 | 3 | 6 | 5 | 22 | 17 | 4 |
| **04512d600_04512d777** | TRUE | 4.78 | 1.16 | 3.71 | 0 | 3 | 8 | 2 | 21 | 17 | 6 |
| **05036d128_05036d43** | TRUE | 5.39 | 1.43 | 7.16 | 1 | 1 | 5 | 5 | 12 | 18 | 15 |
| **04948d250_04948d133** | TRUE | 6.00 | 0.99 | 12.84 | 0 | 0 | 0 | 1 | 10 | 28 | 18 |
| **04876d123_04876d332** | TRUE | 6.17 | 1.15 | 14.73 | 1 | 0 | 0 | 1 | 9 | 24 | 22 |
| **05232d114_05232d156** | TRUE | 6.49 | 1.36 | 18.57 | 0 | 2 | 2 | 1 | 4 | 20 | 28 |
| **04297d338_04297d462** | TRUE | 6.56 | 1.59 | 19.40 | 2 | 1 | 0 | 2 | 8 | 14 | 30 |
| **04237d170_04237d284** | TRUE | 6.59 | 1.18 | 19.79 | 0 | 1 | 0 | 1 | 5 | 20 | 30 |

|  |  |
| --- | --- |

FIGURE S8 **Left panel**: Relative likelihood of observing a given latent value for each mated (light blue curves) or nonmated (light red curves) comparison for the 2018 and 2022 ENFSI data for Examiners when turning off shrinkage. Shrinkage has been turned off making the model free to fit the data without any constraint, of the standard deviation leading to wider standard deviation in some cases.

**Right panel**: Likelihood ratio values for different values along the latent axis for the 2018 and 2022 ENFSI data for Examiners. The likelihood ratio values for different values along the latent axis exhibit a wider range without shrinkage. This is because the model is not being regularized towards a more constrained set of parameter values.

TABLE S7 Data from the 2021 European Network of Forensic Science Institutes investigation for Examiners when turning off shrinkage. We calculated the µ and σ value using the ordered probit model, and sorted the pairs from the lowest µ to the highest µ. The numbers on the right side of the table represent the number of examiners who responded with “Extremely Strong Support for Different People” (m5) , “Very Strong Support for Different People” (m4), “Strong Support for Different People” (m3), “Support for Different People” (m2), and “Weak Support for Different People” (m1), “Inconclusive” (zero), “Weak Support for Same Person” (p1), “Support for Same Person” (p2), “Strong Support for Same Person” (p3), “Very Strong Support for Same Person” (p4), and “Extremely Strong Support for Same Person” (p5). Each pair's ground truth is indicated by the column “Mated” with False referring to nonmated pairs and True referring to mated pairs. We observe wider standard deviation in some cases as well as complex changes in likelihood rations, increasing some while decreasing others.

| **pairID** | **Mated** | **mu** | **sigma** | **LR** | **Proportion Examiners Choosing a Higher Verbal Equivalency Than The LR for LR >1** | **m5** | **m4** | **m3** | **m2** | **m1** | **zero** | **p1** | **p2** | **p3** | **p4** | **p5** |
| --- | --- | --- | --- | --- | --- | --- | --- | --- | --- | --- | --- | --- | --- | --- | --- | --- |
| **C14NM** | FALSE | 2.23 | 1.73 | 0.12 |  | 19 | 16 | 14 | 8 | 2 | 0 | 0 | 2 | 0 | 0 | 0 |
| **C13NM** | FALSE | 2.54 | 1.34 | 0.14 |  | 14 | 15 | 16 | 12 | 4 | 0 | 0 | 0 | 0 | 0 | 0 |
| **C18NM** | FALSE | 3.24 | 1.25 | 0.20 |  | 6 | 12 | 13 | 20 | 7 | 3 | 0 | 0 | 0 | 0 | 0 |
| **C11NM** | FALSE | 3.28 | 1.35 | 0.21 |  | 5 | 13 | 18 | 13 | 3 | 8 | 1 | 0 | 0 | 0 | 0 |
| **C4NM** | FALSE | 3.36 | 2.00 | 0.22 |  | 8 | 11 | 19 | 8 | 7 | 1 | 1 | 3 | 3 | 0 | 0 |
| **C20NM** | FALSE | 3.78 | 1.48 | 0.29 |  | 4 | 10 | 8 | 17 | 12 | 5 | 3 | 2 | 0 | 0 | 0 |
| **C5NM** | FALSE | 3.85 | 1.87 | 0.30 |  | 3 | 13 | 14 | 10 | 8 | 2 | 5 | 3 | 3 | 0 | 0 |
| **C8NM** | FALSE | 4.56 | 1.11 | 0.50 |  | 0 | 3 | 8 | 13 | 14 | 15 | 6 | 2 | 0 | 0 | 0 |
| **C16NM** | FALSE | 4.98 | 1.28 | 0.70 |  | 1 | 0 | 8 | 6 | 16 | 13 | 10 | 6 | 1 | 0 | 0 |
| **C3M** | TRUE | 5.21 | 1.75 | 0.85 |  | 1 | 2 | 5 | 13 | 7 | 10 | 10 | 7 | 5 | 1 | 0 |
| **C12M** | TRUE | 5.57 | 2.59 | 1.15 | 0.49 | 3 | 3 | 6 | 10 | 3 | 6 | 8 | 9 | 10 | 0 | 3 |
| **C6M** | TRUE | 6.00 | 1.47 | 1.66 | 0.57 | 0 | 0 | 4 | 1 | 8 | 13 | 13 | 15 | 6 | 0 | 1 |
| **C2M** | TRUE | 6.50 | 1.92 | 2.52 | 0.57 | 1 | 0 | 3 | 6 | 2 | 5 | 9 | 18 | 14 | 2 | 1 |
| **C15NM** | FALSE | 6.54 | 1.71 | 2.60 | 0.52 | 0 | 1 | 1 | 2 | 4 | 12 | 9 | 18 | 11 | 1 | 2 |
| **C1M** | TRUE | 6.79 | 2.04 | 3.17 | 0.59 | 0 | 0 | 5 | 5 | 1 | 5 | 9 | 13 | 16 | 6 | 1 |
| **C17M** | TRUE | 7.19 | 1.70 | 4.26 | 0.67 | 0 | 0 | 1 | 2 | 3 | 1 | 13 | 20 | 11 | 9 | 1 |
| **C19M** | TRUE | 7.19 | 1.53 | 4.27 | 0.72 | 0 | 0 | 1 | 1 | 1 | 5 | 9 | 23 | 14 | 6 | 1 |
| **C10M** | TRUE | 8.27 | 2.41 | 8.22 | 0.79 | 0 | 1 | 1 | 3 | 1 | 2 | 5 | 7 | 17 | 16 | 8 |
| **C7M** | TRUE | 8.63 | 1.78 | 9.98 | 0.92 | 0 | 0 | 1 | 1 | 0 | 1 | 2 | 8 | 25 | 16 | 7 |
| **C9M** | TRUE | 9.00 | 2.07 | 12.26 | 0.8 | 0 | 1 | 1 | 0 | 0 | 0 | 2 | 8 | 18 | 20 | 11 |

**Wide Mu Prior**

Figure S9**. Left panel:** Relative likelihood of observing a given latent value for each mated (light blue curves) or nonmated (light red curves) comparison for the NIST data with a wide mu prior. The change of prior implies a wider prior distribution for the means. This increased uncertainty led to more flexible and adaptive fitting of the data, allowing the model to be less constrained by prior beliefs when estimating the parameters.

**Right panel**: Likelihood ratio values for different values along the latent axis for the NIST data. The likelihood ratio curve becomes more variable, reflecting the increased uncertainty introduced by the wider priors.

TABLE S8 Data from the Facial examiners from the NIST data with a wide mu prior. We calculated the µ and σ value using the ordered probit model, and sorted the pairs from the lowest µ to the highest µ. The numbers on the right side of the table represent the number of examiners who responded with “strongly support that it is not the same person” (-3), “support that it is not the same person” (-2), and “support to some extent that it is not the same person” (-1), “Support neither that it is the same person nor that it is different persons ” (0), “Support to some extent that it is the same Person” (+1), “support for same person” (+2), “strong support for same Person” (+3). Each pair's ground truth is indicated by the column “Mated” with False referring to nonmated (different source) pairs and True referring to mated (same source) pairs. We observe a slight increase in likelihood ratios, reflecting the increased uncertainty introduced by the wider priors.

| **pairID** | **Mated** | **mu** | **sigma** | **LR** | **m3** | **m2** | **m1** | **zero** | **p1** | **p2** | **p3** |
| --- | --- | --- | --- | --- | --- | --- | --- | --- | --- | --- | --- |
| **04628d459_05252d168** | FALSE | 1.43 | 1.65 | 0.07 | 28 | 19 | 7 | 0 | 1 | 1 | 1 |
| **04509d590_05152d73** | FALSE | 1.98 | 1.65 | 0.14 | 19 | 24 | 9 | 0 | 0 | 4 | 1 |
| **05041d80_04986d102** | FALSE | 2.35 | 1.58 | 0.21 | 14 | 23 | 11 | 2 | 2 | 4 | 1 |
| **04605d377_05017d229** | FALSE | 2.71 | 1.51 | 0.31 | 12 | 17 | 9 | 5 | 11 | 3 | 0 |
| **05115d184_04778d77** | FALSE | 2.75 | 1.12 | 0.32 | 10 | 11 | 17 | 15 | 4 | 0 | 0 |
| **04605d295_05040d47** | FALSE | 2.75 | 1.72 | 0.32 | 11 | 20 | 13 | 0 | 4 | 8 | 1 |
| **04884d76_05180d63** | FALSE | 2.81 | 0.91 | 0.34 | 4 | 19 | 19 | 13 | 1 | 1 | 0 |
| **04670d374_04738d125** | FALSE | 2.97 | 1.27 | 0.41 | 6 | 17 | 16 | 4 | 11 | 3 | 0 |
| **04900d172_04900d155** | TRUE | 3.70 | 0.91 | 0.89 | 2 | 4 | 8 | 22 | 19 | 2 | 0 |
| **04937d81_04937d171** | TRUE | 3.90 | 1.35 | 1.11 | 4 | 4 | 9 | 8 | 25 | 5 | 2 |
| **05113d201_05113d21** | TRUE | 4.39 | 1.14 | 1.89 | 1 | 3 | 4 | 9 | 25 | 14 | 1 |
| **04379d484_04379d636** | TRUE | 4.46 | 1.35 | 2.04 | 1 | 6 | 6 | 2 | 22 | 18 | 2 |
| **05244d182_05244d77** | TRUE | 4.67 | 1.25 | 2.60 | 0 | 3 | 6 | 5 | 22 | 17 | 4 |
| **04512d600_04512d777** | TRUE | 4.78 | 1.36 | 2.91 | 0 | 3 | 8 | 2 | 21 | 17 | 6 |
| **05036d128_05036d43** | TRUE | 5.39 | 1.69 | 5.89 | 1 | 1 | 5 | 5 | 12 | 18 | 15 |
| **04948d250_04948d133** | TRUE | 6.00 | 1.13 | 12.28 | 0 | 0 | 0 | 1 | 10 | 28 | 18 |
| **04876d123_04876d332** | TRUE | 6.16 | 1.48 | 14.88 | 1 | 0 | 0 | 1 | 9 | 24 | 22 |
| **05232d114_05232d156** | TRUE | 6.49 | 1.77 | 22.20 | 0 | 2 | 2 | 1 | 4 | 20 | 28 |
| **04297d338_04297d462** | TRUE | 6.58 | 2.12 | 24.83 | 2 | 1 | 0 | 2 | 8 | 14 | 30 |
| **04237d170_04237d284** | TRUE | 6.64 | 1.53 | 26.65 | 0 | 1 | 0 | 1 | 5 | 20 | 30 |

|  |  |
| --- | --- |

FIGURE S10 **Left panel:** Relative likelihood of observing a given latent value for each mated (light blue curves) or nonmated (light red curves) comparison for the 2018 and 2022 ENFSI data for Examiners. The change of prior implies a wider prior distribution for the means. This increased uncertainty led to more flexible and adaptive fitting of the data, allowing the model to be less constrained by prior beliefs when estimating the parameters.

**Right panel**: Likelihood ratio values for different values along the latent axis for the 2018 and 2022 ENFSI data for Examiners. The likelihood ratio curve becomes more variable, reflecting the increased uncertainty introduced by the wider priors.

TABLE S9 Data from the 2021 European Network of Forensic Science Institutes investigation for Examiners with a wide mu prior. We calculated the µ and σ value using the ordered probit model, and sorted the pairs from the lowest µ to the highest µ. The numbers on the right side of the table represent the number of examiners who responded with “Extremely Strong Support for Different People” (m5) , “Very Strong Support for Different People” (m4), “Strong Support for Different People” (m3), “Support for Different People” (m2), and “Weak Support for Different People” (m1), “Inconclusive” (zero), “Weak Support for Same Person” (p1), “Support for Same Person” (p2), “Strong Support for Same Person” (p3), “Very Strong Support for Same Person” (p4), and “Extremely Strong Support for Same Person” (p5). Each pair's ground truth is indicated by the column “Mated” with False referring to nonmated pairs and True referring to mated pairs. We observe a slight increase in likelihood ratios, reflecting the increased uncertainty introduced by the wider priors.

| **pairID** | **Mated** | **mu** | **sigma** | **LR** | **Proportion Examiners Choosing a Higher Verbal Equivalency Than The LR for LR > 1** | **m5** | **m4** | **m3** | **m2** | **m1** | **zero** | **p1** | **p2** | **p3** | **p4** | **p5** |
| --- | --- | --- | --- | --- | --- | --- | --- | --- | --- | --- | --- | --- | --- | --- | --- | --- |
| **C14NM** | FALSE | 2.27 | 1.72 | 0.10 |  | 19 | 16 | 14 | 8 | 2 | 0 | 0 | 2 | 0 | 0 | 0 |
| **C13NM** | FALSE | 2.57 | 1.41 | 0.12 |  | 14 | 15 | 16 | 12 | 4 | 0 | 0 | 0 | 0 | 0 | 0 |
| **C18NM** | FALSE | 3.29 | 1.33 | 0.19 |  | 6 | 12 | 13 | 20 | 7 | 3 | 0 | 0 | 0 | 0 | 0 |
| **C11NM** | FALSE | 3.33 | 1.41 | 0.20 |  | 5 | 13 | 18 | 13 | 3 | 8 | 1 | 0 | 0 | 0 | 0 |
| **C4NM** | FALSE | 3.41 | 1.93 | 0.21 |  | 8 | 11 | 19 | 8 | 7 | 1 | 1 | 3 | 3 | 0 | 0 |
| **C20NM** | FALSE | 3.84 | 1.52 | 0.29 |  | 4 | 10 | 8 | 17 | 12 | 5 | 3 | 2 | 0 | 0 | 0 |
| **C5NM** | FALSE | 3.90 | 1.83 | 0.30 |  | 3 | 13 | 14 | 10 | 8 | 2 | 5 | 3 | 3 | 0 | 0 |
| **C8NM** | FALSE | 4.64 | 1.17 | 0.52 |  | 0 | 3 | 8 | 13 | 14 | 15 | 6 | 2 | 0 | 0 | 0 |
| **C16NM** | FALSE | 5.06 | 1.33 | 0.72 |  | 1 | 0 | 8 | 6 | 16 | 13 | 10 | 6 | 1 | 0 | 0 |
| **C3M** | TRUE | 5.28 | 1.71 | 0.87 |  | 1 | 2 | 5 | 13 | 7 | 10 | 10 | 7 | 5 | 1 | 0 |
| **C12M** | TRUE | 5.63 | 2.35 | 1.16 | 0.49 | 3 | 3 | 6 | 10 | 3 | 6 | 8 | 9 | 10 | 0 | 3 |
| **C6M** | TRUE | 6.07 | 1.48 | 1.68 | 0.57 | 0 | 0 | 4 | 1 | 8 | 13 | 13 | 15 | 6 | 0 | 1 |
| **C2M** | TRUE | 6.56 | 1.85 | 2.50 | 0.57 | 1 | 0 | 3 | 6 | 2 | 5 | 9 | 18 | 14 | 2 | 1 |
| **C15NM** | FALSE | 6.59 | 1.67 | 2.58 | 0.52 | 0 | 1 | 1 | 2 | 4 | 12 | 9 | 18 | 11 | 1 | 2 |
| **C1M** | TRUE | 6.84 | 1.93 | 3.12 | 0.59 | 0 | 0 | 5 | 5 | 1 | 5 | 9 | 13 | 16 | 6 | 1 |
| **C17M** | TRUE | 7.24 | 1.65 | 4.20 | 0.67 | 0 | 0 | 1 | 2 | 3 | 1 | 13 | 20 | 11 | 9 | 1 |
| **C19M** | TRUE | 7.24 | 1.52 | 4.21 | 0.72 | 0 | 0 | 1 | 1 | 1 | 5 | 9 | 23 | 14 | 6 | 1 |
| **C10M** | TRUE | 8.29 | 2.19 | 8.19 | 0.79 | 0 | 1 | 1 | 3 | 1 | 2 | 5 | 7 | 17 | 16 | 8 |
| **C7M** | TRUE | 8.65 | 1.72 | 10.12 | 0.79 | 0 | 0 | 1 | 1 | 0 | 1 | 2 | 8 | 25 | 16 | 7 |
| **C9M** | TRUE | 9.00 | 1.94 | 12.41 | 0.8 | 0 | 1 | 1 | 0 | 0 | 0 | 2 | 8 | 18 | 20 | 11 |

**T-distribution**

FIGURE S11 **Left panel**: Relative likelihood of observing a given latent value for each mated (light blue curves) or nonmated (light red curves) comparison for the NIST data. We use a T-distribution instead of a normal distribution. We can see that the t-distribution has fatter tails compared to the normal distribution.

**Right panel**: Likelihood ratio values for different values along the latent axis for the NIST data. The t-distribution results in differences in the shape and values of the likelihood ratio curve compared to using a normal distribution. In the case of the t-distribution, the likelihood ratio is influenced by the heavy tails, leading to a peak value before the probabilities start to decrease as you move further away from the mean. The non-monotonic relation between μ and the likelihood ratios at both ends of the scale suggest that the t-distribution is not a tenable assumption in the ordered probit model.

TABLE S10 Data from the Facial examiners from the NIST data using a T-distribution. We calculated the µ and σ value using the ordered probit model, and sorted the pairs from the lowest µ to the highest µ. The numbers on the right side of the table represent the number of examiners who responded with “strongly support that it is not the same person” (-3), “support that it is not the same person” (-2), and “support to some extent that it is not the same person” (-1), “Support neither that it is the same person nor that it is different persons ” (0), “Support to some extent that it is the same Person” (+1), “support for same person” (+2), “strong support for same Person” (+3). Each pair's ground truth is indicated by the column “Mated” with False referring to nonmated (different source) pairs and True referring to mated (same source) pairs. We observe lower likelihood ratios due to being influenced by the heavy tails.

| **pairID** | **Mated** | **mu** | **sigma** | **LR** | **m3** | **m2** | **m1** | **zero** | **p1** | **p2** | **p3** |
| --- | --- | --- | --- | --- | --- | --- | --- | --- | --- | --- | --- |
| **04628d459_05252d168** | FALSE | 1.49 | 1.20 | 0.07 | 28 | 19 | 7 | 0 | 1 | 1 | 1 |
| **04509d590_05152d73** | FALSE | 1.92 | 1.20 | 0.10 | 19 | 24 | 9 | 0 | 0 | 4 | 1 |
| **05041d80_04986d102** | FALSE | 2.27 | 1.23 | 0.13 | 14 | 23 | 11 | 2 | 2 | 4 | 1 |
| **04605d295_05040d47** | FALSE | 2.64 | 1.41 | 0.20 | 11 | 20 | 13 | 0 | 4 | 8 | 1 |
| **04605d377_05017d229** | FALSE | 2.73 | 1.33 | 0.22 | 12 | 17 | 9 | 5 | 11 | 3 | 0 |
| **04884d76_05180d63** | FALSE | 2.87 | 0.79 | 0.27 | 4 | 19 | 19 | 13 | 1 | 1 | 0 |
| **05115d184_04778d77** | FALSE | 2.88 | 1.00 | 0.28 | 10 | 11 | 17 | 15 | 4 | 0 | 0 |
| **04670d374_04738d125** | FALSE | 2.98 | 1.10 | 0.32 | 6 | 17 | 16 | 4 | 11 | 3 | 0 |
| **04900d172_04900d155** | TRUE | 3.78 | 0.72 | 1.06 | 2 | 4 | 8 | 22 | 19 | 2 | 0 |
| **04937d81_04937d171** | TRUE | 3.99 | 1.08 | 1.42 | 4 | 4 | 9 | 8 | 25 | 5 | 2 |
| **05113d201_05113d21** | TRUE | 4.45 | 0.94 | 2.54 | 1 | 3 | 4 | 9 | 25 | 14 | 1 |
| **04379d484_04379d636** | TRUE | 4.57 | 1.14 | 2.91 | 1 | 6 | 6 | 2 | 22 | 18 | 2 |
| **05244d182_05244d77** | TRUE | 4.69 | 1.06 | 3.34 | 0 | 3 | 6 | 5 | 22 | 17 | 4 |
| **04512d600_04512d777** | TRUE | 4.78 | 1.16 | 3.71 | 0 | 3 | 8 | 2 | 21 | 17 | 6 |
| **05036d128_05036d43** | TRUE | 5.39 | 1.43 | 7.16 | 1 | 1 | 5 | 5 | 12 | 18 | 15 |
| **04948d250_04948d133** | TRUE | 6.00 | 0.99 | 12.84 | 0 | 0 | 0 | 1 | 10 | 28 | 18 |
| **04876d123_04876d332** | TRUE | 6.17 | 1.15 | 14.73 | 1 | 0 | 0 | 1 | 9 | 24 | 22 |
| **05232d114_05232d156** | TRUE | 6.49 | 1.36 | 18.57 | 0 | 2 | 2 | 1 | 4 | 20 | 28 |
| **04297d338_04297d462** | TRUE | 6.56 | 1.59 | 19.40 | 2 | 1 | 0 | 2 | 8 | 14 | 30 |
| **04237d170_04237d284** | TRUE | 6.59 | 1.18 | 19.79 | 0 | 1 | 0 | 1 | 5 | 20 | 30 |

|  |  |
| --- | --- |

FIGURE S12 **Left panel**: Relative likelihood of observing a given latent value for each mated (light blue curves) or nonmated (light red curves) comparison for the 2018 and 2022 ENFSI data for Examiners. We use a T-distribution instead of a normal distribution. We can see that the t-distribution has fatter tails compared to the normal distribution.

**Right panel**: Likelihood ratio values for different values along the latent axis for the 2018 and 2022 ENFSI data for Examiners. The t-distribution results in differences in the shape and values of the likelihood ratio curve compared to using a normal distribution. In the case of the t-distribution, the likelihood ratio is influenced by the heavy tails, leading to a peak value before the probabilities start to decrease as you move further away from the mean. The non-monotonic relation between μ and the likelihood ratios at both ends of the scale suggest that the t-distribution is not a tenable assumption in the ordered probit model.

TABLE S11 Data from the 2021 European Network of Forensic Science Institutes investigation for Examiners using a T-distribution. We calculated the µ and σ value using the ordered probit model, and sorted the pairs from the lowest µ to the highest µ. The numbers on the right side of the table represent the number of examiners who responded with “Extremely Strong Support for Different People” (m5) , “Very Strong Support for Different People” (m4), “Strong Support for Different People” (m3), “Support for Different People” (m2), and “Weak Support for Different People” (m1), “Inconclusive” (zero), “Weak Support for Same Person” (p1), “Support for Same Person” (p2), “Strong Support for Same Person” (p3), “Very Strong Support for Same Person” (p4), and “Extremely Strong Support for Same Person” (p5). Each pair's ground truth is indicated by the column “Mated” with False referring to nonmated pairs and True referring to mated pairs. We observe lower likelihood ratios due to being influenced by the heavy tails.

| **pairID** | **Mated** | **mu** | **sigma** | **LR** | **Proportion Examiners Choosing a Higher Verbal Equivalency Than The LR for LR >1** | **m5** | **m4** | **m3** | **m2** | **m1** | **zero** | **p1** | **p2** | **p3** | **p4** | **p5** |
| --- | --- | --- | --- | --- | --- | --- | --- | --- | --- | --- | --- | --- | --- | --- | --- | --- |
| **C14NM** | FALSE | 2.30 | 1.44 | 0.10 |  | 19 | 16 | 14 | 8 | 2 | 0 | 0 | 2 | 0 | 0 | 0 |
| **C13NM** | FALSE | 2.67 | 1.30 | 0.11 |  | 14 | 15 | 16 | 12 | 4 | 0 | 0 | 0 | 0 | 0 | 0 |
| **C4NM** | FALSE | 3.33 | 1.54 | 0.16 |  | 8 | 11 | 19 | 8 | 7 | 1 | 1 | 3 | 3 | 0 | 0 |
| **C11NM** | FALSE | 3.39 | 1.25 | 0.17 |  | 5 | 13 | 18 | 13 | 3 | 8 | 1 | 0 | 0 | 0 | 0 |
| **C18NM** | FALSE | 3.44 | 1.19 | 0.18 |  | 6 | 12 | 13 | 20 | 7 | 3 | 0 | 0 | 0 | 0 | 0 |
| **C5NM** | FALSE | 3.82 | 1.53 | 0.24 |  | 3 | 13 | 14 | 10 | 8 | 2 | 5 | 3 | 3 | 0 | 0 |
| **C20NM** | FALSE | 3.98 | 1.31 | 0.27 |  | 4 | 10 | 8 | 17 | 12 | 5 | 3 | 2 | 0 | 0 | 0 |
| **C8NM** | FALSE | 4.75 | 1.03 | 0.52 |  | 0 | 3 | 8 | 13 | 14 | 15 | 6 | 2 | 0 | 0 | 0 |
| **C16NM** | FALSE | 5.16 | 1.11 | 0.75 |  | 1 | 0 | 8 | 6 | 16 | 13 | 10 | 6 | 1 | 0 | 0 |
| **C3M** | TRUE | 5.30 | 1.41 | 0.85 |  | 1 | 2 | 5 | 13 | 7 | 10 | 10 | 7 | 5 | 1 | 0 |
| **C12M** | TRUE | 5.63 | 1.90 | 1.17 | 0.49 | 3 | 3 | 6 | 10 | 3 | 6 | 8 | 9 | 10 | 0 | 3 |
| **C6M** | TRUE | 6.07 | 1.18 | 1.73 | 0.57 | 0 | 0 | 4 | 1 | 8 | 13 | 13 | 15 | 6 | 0 | 1 |
| **C15NM** | FALSE | 6.56 | 1.32 | 2.55 | 0.52 | 0 | 1 | 1 | 2 | 4 | 12 | 9 | 18 | 11 | 1 | 2 |
| **C2M** | TRUE | 6.68 | 1.48 | 2.78 | 0.57 | 1 | 0 | 3 | 6 | 2 | 5 | 9 | 18 | 14 | 2 | 1 |
| **C1M** | TRUE | 6.92 | 1.61 | 3.30 | 0.59 | 0 | 0 | 5 | 5 | 1 | 5 | 9 | 13 | 16 | 6 | 1 |
| **C17M** | TRUE | 7.20 | 1.36 | 3.98 | 0.67 | 0 | 0 | 1 | 2 | 3 | 1 | 13 | 20 | 11 | 9 | 1 |
| **C19M** | TRUE | 7.21 | 1.23 | 4.01 | 0.72 | 0 | 0 | 1 | 1 | 1 | 5 | 9 | 23 | 14 | 6 | 1 |
| **C10M** | TRUE | 8.42 | 1.77 | 8.79 | 0.79 | 0 | 1 | 1 | 3 | 1 | 2 | 5 | 7 | 17 | 16 | 8 |
| **C7M** | TRUE | 8.69 | 1.36 | 10.40 | 0.79 | 0 | 0 | 1 | 1 | 0 | 1 | 2 | 8 | 25 | 16 | 7 |
| **C9M** | TRUE | 9.07 | 1.49 | 12.96 | 0.8 | 0 | 1 | 1 | 0 | 0 | 0 | 2 | 8 | 18 | 20 | 11 |


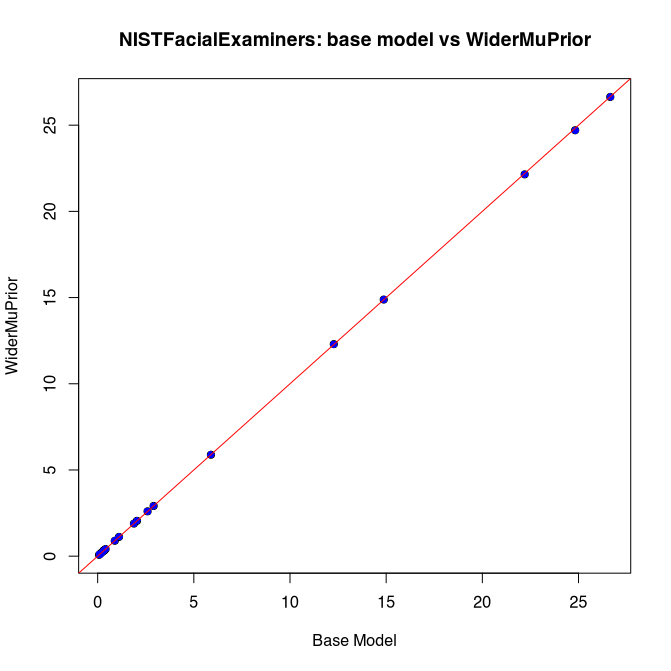


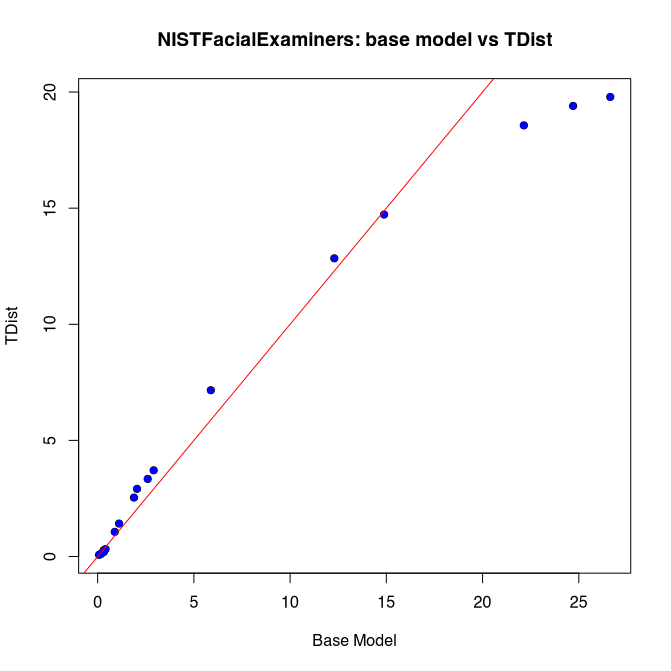

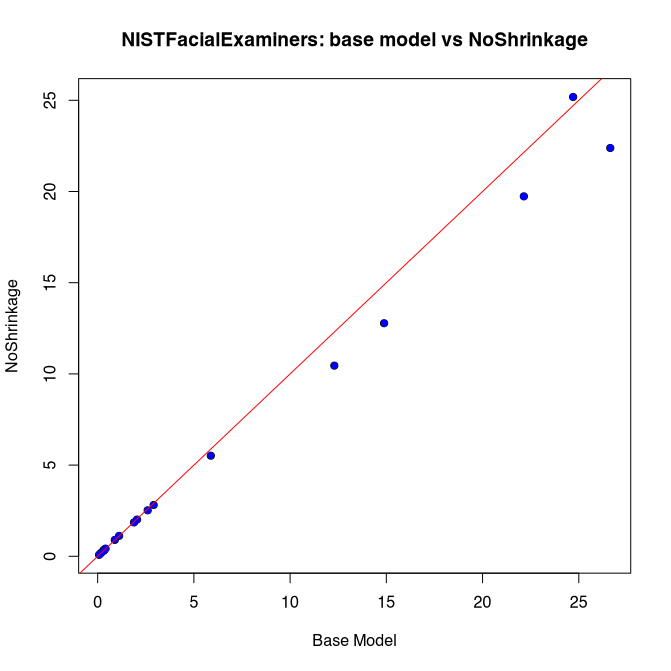


FIGURE S13 Scatterplot showing a direct comparisons between different versions of the ordered probit model, and exploring a wider range of values for the priors. Notice that none of the changes (increasing the mu value, using a T-distribution and turning off shrinkage) produce substantive shifts in the likelihood ratios. This is primarily because the large datasets tend to quickly overwhelm the priors.

## NIST Data Super Recognizers Performances

|  |  |
| --- | --- |

FIGURE S14 **Left panel**: Relative likelihood of observing a given latent value for each mated (light blue curves) or nonmated (light red curves) comparison for the super-recognizers in the NIST data. The parameters for each normal distribution were derived from the ordered probit model fit to all eleven conclusions for each comparison. The thick red curve corresponds to the sum of light red curves. It represents the relative likelihood of observing any nonmated comparison at each value of the latent axis. The thick blue curve represents the relative likelihood of observing any mated comparison at each value of the latent axis while the thick red curve represents the relative likelihood of observing any nonmated comparison at each value of the latent axis. The vertical lines correspond to the median threshold values that divide the latent axis to produce the estimate of the proportion of responses in each bin. P-labels correspond to positive support statements and m-labels correspond to negative support statements (support for different persons).

**Right panel**: Likelihood ratio values for different values along the latent axis for the super-recognizers in the NIST data. This curve is the ratio of the thick blue divided by the thick red curve in the left panel. The y axis is plotted on a log(10) axis. Likelihood ratios for individual mated pairs are shown as blue circles, and likelihood ratios for nonmated pairs are shown as red circles.

TABLE S12 Data from super-recognizers in the NIST data. We calculated the µ and σ value using the ordered probit model, and sorted the pairs from the lowest µ to the highest µ. The numbers on the right side of the table represent the number of examiners who responded with “strong support that it is the same person” (p3) , “support that it is the same person” (p2), “support to some extent that it is the same person (p1), “support neither that it is the same person nor that it is different persons (zero), and “support to some extent that it is not the same person” (m1), “support that it is not the same person” (m2), “strong support that it is not the same person” (m3). Each pair's ground truth is indicated by the column “Mated” with False referring to nonmated pairs and True referring to mated pairs. We observe lower likelihood ratios due to being influenced by the heavy tails.

| **pairID** | **Mated** | **mu** | **sigma** | **LR** | **m3** | **m2** | **m1** | **zero** | **p1** | **p2** | **p3** |
| --- | --- | --- | --- | --- | --- | --- | --- | --- | --- | --- | --- |
| **04884d76_05180d63** | FALSE | 1.03 | 2.31 | 0.21 | 7 | 5 | 0 | 0 | 1 | 0 | 0 |
| **04628d459_05252d168** | FALSE | 1.69 | 2.18 | 0.30 | 5 | 6 | 2 | 0 | 0 | 0 | 0 |
| **05115d184_04778d77** | FALSE | 1.74 | 2.22 | 0.31 | 5 | 6 | 1 | 0 | 1 | 0 | 0 |
| **04605d295_05040d47** | FALSE | 1.76 | 2.44 | 0.31 | 6 | 4 | 1 | 0 | 0 | 2 | 0 |
| **04670d374_04738d125** | FALSE | 1.92 | 2.43 | 0.34 | 5 | 6 | 1 | 0 | 0 | 0 | 1 |
| **04509d590_05152d73** | FALSE | 2.28 | 2.40 | 0.41 | 5 | 3 | 3 | 0 | 0 | 2 | 0 |
| **04605d377_05017d229** | FALSE | 2.51 | 2.17 | 0.46 | 3 | 6 | 3 | 0 | 0 | 1 | 0 |
| **05041d80_04986d102** | FALSE | 2.74 | 2.35 | 0.53 | 4 | 3 | 2 | 1 | 1 | 2 | 0 |
| **05036d128_05036d43** | TRUE | 3.69 | 2.39 | 0.91 | 3 | 2 | 1 | 0 | 1 | 6 | 0 |
| **04379d484_04379d636** | TRUE | 4.51 | 2.43 | 1.49 | 2 | 1 | 2 | 0 | 2 | 4 | 2 |
| **04937d81_04937d171** | TRUE | 5.03 | 2.49 | 2.07 | 1 | 2 | 3 | 0 | 1 | 2 | 4 |
| **05244d182_05244d77** | TRUE | 5.07 | 2.19 | 2.12 | 0 | 3 | 1 | 0 | 1 | 6 | 2 |
| **05232d114_05232d156** | TRUE | 5.16 | 2.52 | 2.24 | 1 | 3 | 1 | 0 | 0 | 4 | 4 |
| **04876d123_04876d332** | TRUE | 5.43 | 2.44 | 2.65 | 1 | 1 | 2 | 0 | 1 | 4 | 4 |
| **04900d172_04900d155** | TRUE | 5.52 | 2.34 | 2.81 | 0 | 3 | 0 | 1 | 1 | 4 | 4 |
| **05113d201_05113d21** | TRUE | 6.06 | 2.27 | 4.00 | 0 | 1 | 0 | 0 | 4 | 3 | 5 |
| **04297d338_04297d462** | TRUE | 6.14 | 2.30 | 4.21 | 0 | 1 | 2 | 0 | 0 | 5 | 5 |
| **04512d600_04512d777** | TRUE | 6.36 | 2.57 | 4.86 | 0 | 3 | 1 | 0 | 0 | 2 | 7 |
| **04948d250_04948d133** | TRUE | 6.43 | 2.56 | 5.10 | 0 | 3 | 0 | 0 | 1 | 2 | 7 |
| **04237d170_04237d284** | TRUE | 8.05 | 2.58 | 15.49 | 0 | 1 | 1 | 0 | 0 | 1 | 10 |

## Correlation Between NIST Examiners Performance and Algorithm A2017b

FIGURE S15 Scatterplot showing the relationship between the Logarithmic Likelihood Ratio (log(LR)) decisions of NIST forensic facial examiners and the similarity scores produced by the deep convolutional neural network A2017b. Each point represents a face-pair judgment, with the red regression line indicating a positive correlation (*r* = 0.540). Consistent with Phillips, Yates [5], A2017b achieved performance exceeding the median of forensic examiners, and the modest correlation here reflects that while the algorithm and human experts capture overlapping information, their decision strategies are not identical.

**Summing the Normal Curves**

Although relatively novel in the literature, the ordered probit model likelihood ratios we use in the paper are based on the foundations of signal detection theory, one of the most widely-used approach in psychological science. The assumption of adding the normal distributions to create the overall mated and nonmated distributions is predicated on the simple ‘or’ rule in probability. We have created a brief video explaining why we add normal distributions to create the overall mated and nonmated distributions:

[https://iu.mediaspace.kaltura.com/media/t/1_69n0s1ny](https://nam12.safelinks.protection.outlook.com/?url=https%3A%2F%2Fiu.mediaspace.kaltura.com%2Fmedia%2Ft%2F1_69n0s1ny&data=05%7C02%7Cnaggadi%40iu.edu%7C8e5229d4858c42c81efa08de21554e08%7C1113be34aed14d00ab4bcdd02510be91%7C1%7C0%7C638984847424893204%7CUnknown%7CTWFpbGZsb3d8eyJFbXB0eU1hcGkiOnRydWUsIlYiOiIwLjAuMDAwMCIsIlAiOiJXaW4zMiIsIkFOIjoiTWFpbCIsIldUIjoyfQ%3D%3D%7C0%7C%7C%7C&sdata=7a3LiKaAdHHmdY5ToYtjTYIY5lBw1gTm0xQP3v7drYs%3D&reserved=0)

We are essentially saying is: What is the probability that a given latent value came from ANY mated pair (i.e. Pair 1 OR Pair 2 OR Pair 3, etc.). This logic allows us to sum over the normal distributions. When reference databases are created using machine-based similarity in domains such as firearms, similarity scores for mated and non-mated pairs are collected into distributions and normalized to compute the probability of observing a particular similarity score from a mated or non-mated pair. Our approach is no different than this, except that rather than summing together (or counting) individual scores at each level of similarity, we are summing normal distributions at each point along of the latent dimension. The facial comparison experts act as our similarity computation, and the normal distribution summarizes the set of latent scores that must have been obtained in the minds of examiners to produce the set of verbal responses we observe for a given image pair.

It is possible to approximate the overall mated and nonmated distributions without the addition of the normal distribution curves. We simply need to aggregate the raw scores across all mated and nonmated pairs. For example, the NIST facial examiner table looks like this:

| pairID | Mated | m3 | m2 | m1 | zero | p1 | p2 | p3 |
| --- | --- | --- | --- | --- | --- | --- | --- | --- |
| All_Nonmated_Combined | FALSE | 104 | 150 | 101 | 39 | 34 | 24 | 4 |
| All_Mated_Combined | TRUE | 12 | 28 | 48 | 59 | 182 | 197 | 158 |

When we fit this data with the Ordered Probit Model we have 12 degrees of freedom and 4 estimated thresholds plus 2 means and 2 standard deviations (8 total parameters). There is only one mated and only one nonmated curve as shown below, which are quite similar to the overall curves in Figure 2 in the main paper. The differences come in part due to the fact that the overall curves in Figure 2 are not normally distributed (the sum of normals is not generally a normal distribution) and those below are constrained to be normal distributions. However, the curves in Figure S16 are quite similar to the curves in Figure 2.

The likelihood ratio for the mated pairs in the above analysis is 4.6, which is very close to the median likelihood ratio of 4.4 in the original data (Table 1 in the main paper). Thus we believe that the fits to the aggregate data are quite similar to the fits to individual image pairs that are then summed to create overall mated and nonmated distributions.

Figure 3 in the main paper is the posterior predictive check that compares the area to the right of each threshold to the empirical proportion of responses made by subjects, and is generally in line with the empirical proportions. The aggregated analysis described above produces a very similar posterior predictive check graph as shown in the right panel of Figure S16. Thus we believe that the way that we are combining across normal distributions through summation is logically and mathematically correct since fits to the aggregated data give very similar results without involving summed normal distributions.

FIGURE S16 **Left panel**: Relative likelihood of observing a given latent value for each mated (light blue curves) or nonmated (light red curves) comparison when we aggregate the raw scores across all mated and nonmated pairs. There is only one mated and only one nonmated curve as shown below, which are quite similar to the overall curves in Figure 2 in the main paper. The differences come in part due to the fact that the overall curves in Figure 2 are not normally distributed (the sum of normals is not generally a normal distribution) and those below are constrained to be normal distributions. The likelihood ratio for the mated pairs in the above analysis is 4.6, which is very close to the median likelihood ratio of 4.4 in the original data (Table 1 in the main paper). Thus we believe that the fits to the aggregate data are quite similar to the fits to individual image pairs that are then summed to create overall mated and nonmated distributions.

**Right panel**: posterior predictive check that compares the area to the right of each threshold to the empirical proportion of responses made by subjects when we aggregate the raw scores across all mated and nonmated pairs. This figure is quite similar to the curves in Figure 3 which suggests that the way that we are combining across normal distributions through summation is logically and mathematically correct since fits to the aggregated data give very similar results without involving summed normal distributions.

**References**

1. Wixted JT. The forgotten history of signal detection theory. J Exp Psychol Learn Mem Cogn. 2020;46(2):201–33. doi: 10.1037/xlm0000732

2. Cuellar M, Vanderplas S, Luby A, Rosenblum M. Methodological problems in every black-box study of forensic firearm comparisons. Law Probab Risk. 2024;23(1):mgae015. doi: 10.1093/lpr/mgae015

3. Ideal Innovations Incorporated. Facial identification proficiency tests. 2019. Available from: <https://www.idealinnovations.com/face-center-of-excellence/facial-identification-proficiency-test/>. Accessed 11 Dec 2025.

4. Collaborative Testing Services, Inc. Facial identification examination test no. 24-5571 summary report. 2024. Available from: <https://cts-forensics.com/reports/24-5571_Web.pdf>. Accessed 11 Dec 2025.

5. Phillips PJ, Yates AN, Hu Y, Hahn CA, Noyes E, Jackson K, et al. Face recognition accuracy of forensic examiners, superrecognizers, and face recognition algorithms. Proc Natl Acad Sci USA. 2018;115(24):6171–6. doi: 10.1073/pnas.1721355115
